# Argument-based inductive logics, with coverage of compromised perception

**DOI:** 10.3389/frai.2023.1144569

**Published:** 2024-01-08

**Authors:** Selmer Bringsjord, Michael Giancola, Naveen Sundar Govindarajulu, John Slowik, James Oswald, Paul Bello, Micah Clark

**Affiliations:** ^1^Rensselaer AI & Reasoning (RAIR) Lab, Department of Computer Science, Department of Cognitive Science, Rensselaer Polytechnic Institute, Troy, NY, United States; ^2^Naval Research Laboratory, Washington, DC, United States; ^3^College of Information Sciences and Technology, Pennsylvania State University, State College, PA, United States

**Keywords:** inductive logic, compromised perception, argument and automated reasoning, Monty Hall dilemma, cognitive robotics, AI

## Abstract

Formal deductive logic, used to express and reason over declarative, axiomatizable content, captures, we now know, essentially all of what is known in mathematics and physics, and captures as well the details of the proofs by which such knowledge has been secured. This is certainly impressive, but deductive logic alone cannot enable rational adjudication of arguments that are at variance (however much additional information is added). After affirming a fundamental directive, according to which argumentation should be the basis for human-centric AI, we introduce and employ both a deductive and—crucially—an inductive *cognitive calculus*. The former cognitive calculus, DCEC, is the deductive one and is used with our automated deductive reasoner ShadowProver; the latter, IDCEC, is inductive, is used with the automated inductive reasoner ShadowAdjudicator, and is based on human-used concepts of *likelihood* (and in some dialects of IDCEC, probability). We explain that ShadowAdjudicator centers around the concept of competing and nuanced arguments adjudicated non-monotonically through time. We make things clearer and more concrete by way of three case studies, in which our two automated reasoners are employed. Case Study 1 involves the famous Monty Hall Problem. Case Study 2 makes vivid the efficacy of our calculi and automated reasoners in simulations that involve a cognitive robot (PERI.2). In Case Study 3, as we explain, the simulation employs the cognitive architecture ARCADIA, which is designed to computationally model human-level cognition in ways that take perception and attention seriously. We also discuss a type of argument rarely analyzed in logic-based AI; arguments intended to persuade by leveraging human deficiencies. We end by sharing thoughts about the future of research and associated engineering of the type that we have displayed.

## 1 Introduction

Formal deductive logic, used to express and reason over declarative, axiomatizable content, captures, we now know, essentially all of what is known in mathematics and physics, and captures as well the details of the proofs by which such knowledge has been secured. This is impressive certainly, but even simple scenarios explain a very different story: for example, if (human) Alice perceives a blue cube on a table, then accordingly declares that she believes that there is a blue cube thereon, while Bob, beside her and looking also at the table through his pair of glasses, asserts “No, actually that's an orange sphere,” deductive logic alone cannot enable rational adjudication of the disagreements between them. The great pioneer of modern inductive logic, Rudolph Carnap, fully understood this in the mid-20th century during the heyday period of deductive logic brought about principally by Gödel. Carnap would say, and the logicians and mathematicians today who continue his vibrant legacy in the form of what is known as *pure inductive logic* (PIL) (Paris and Vencovská, 2015) would still say, that “There is a blue cube on the table” and “There is an orange sphere on the table” should each be assigned a probability value (a real number between 0 and 1, inclusive), and this content, combined with additional probabilitized propositions, can be used in a process that dictates what should be rationally believed. Unfortunately, Carnap and his followers pay little attention to the “coin of the realm” in human reasoning and decision-making: arguments and argumentation.^1^ This tradition (which began before Carnap and includes e.g., Keynes, 1921) also runs afoul of the brute fact that humans very rarely use probabilities and the probability calculus (and when they are “boxed in” to using probabilities, very rarely use them correctly, as shown by the infamous “Linda Problem”, nicely discussed in this connection by Kahneman, 2013). In addition, this tradition in inductive logic avoids the brute fact that Alice and Bob, humans in general, and also today's cognitive robots, inevitably perceive in messy environments that render percepts highly uncertain (e.g., what are the ambient lighting conditions in the room Alice and Bob are in?). We introduce below a family of novel inductive logics, based centrally on human-used concepts of *likelihood*, that center around the concept of competing, nuanced arguments adjudicated through time. We present three case studies in which likelihood is key: Case Study 1 involves the famous Monty Hall Problem.^2^ Case Study 2 makes vivid the efficacy of our calculi and automated reasoners in simulations that involve the robot (PERI.2). In Case Study 3, as we explain, the simulation employs automated reasoners joined with the cognitive architecture ARCADIA, which is designed to computationally model cognition in ways that take perception and attention seriously. Penultimately, we discuss a class of arguments hitherto largely ignored in logicist AI, such as arguments designed to persuade despite the fact that they are unsound. We end by sharing thoughts about the future of research and associated engineering of the type that we have displayed herein.

The remainder of the present study unfolds as follows. In the next Section 2, we explain, affirm, and (albeit briefly) defend our “prime directive,” in a word that argumentation must be the basis of human-level, and human-centric, AI. Next, we (Section 3) briefly point out that, putting it mildly, perception has not exactly been treated in a deep way in the history of logicist AI—despite the fact that immediately instructive parables such as the Alice-Bob sketched above have been obvious since McKeon (1941)^3^ presented to humanity, in his *Organon*, the first formal logic, with algorithms for determining whether arguments expressed therein are formally valid.^4^ What follows is a section devoted to giving an historical perspective on our research (Section 4) and coverage of a considerable amount of related prior study. The next section lists the specific desiderata for argument-centric automated defeasible (= non-monotonic) reasoning that we seek and abide by and which are satisfied by the logico-mathematics, systems, and case-study demonstration we present herein. We then (Section 6) orient the reader to our brand of logicist AI by briefly explaining our background logico-mathematics; this section ends with a sub-section in which the specifications for the two pivotal cognitive calculi alluded to above (DCEC & IDCEC) are given. Section 7 presents, in turn, the three case studies we have promised above. The penultimate section of the study is devoted to explaining a category of arguments premeditatedly designed to be unsound but (in fact in some cases more) persuasive. In our final Section 9, we touch upon the need to solve paradoxes in the intersection of reasoning and perception, point out that future study is needed to address pictorial arguments (which are common in the human case), and offer a few final remarks.

## 2 Argumentation must ground human-centric AI

We believe that the basis for rational human use of AI technology is, or at least ought to be, argumentation, computationally treated, and managed. In this regard, we wholly concur with Dietz et al. (2022). For us, this is a firm and fundamental directive that guides our research. For convenient reference to this directive in the remainder of the present study, we refer to it as simply ‘Dir'. Notably, we stipulate that Dir specifies for us *rational* human use of AI. Obviously, there are irrational uses of AI that, by definition, make argumentation decidedly unwanted, for at least some of the humans involved. For instance, Jones may wish to simply make, activate, and then violently destroy AI technology (because he is in the grip of an pathological level of hatred of all things both artificial and human-like), and it is exceedingly hard to observe how this non-cerebral use of AI should be mediated by argumentation.^5^ Of course, we anticipate that most human use of AI technology will indeed be rational.

So far, we have referred to “AI technology.” Let us be a bit more accurate, by speaking of **artificial agents**, in accordance with the comprehensive, respected textbooks for the field of AI (see Luger, 2008; Russell and Norvig, 2020). In these studies, in broad strokes, which suffice for the present study, artificial agents, located in a given environment, take in percepts of that environment as input and compute in some fashion over this input (along with various information from other sources and of other types), and this computation leads them to perform actions as output. In our approach, that of logic-based/logicist AI, the computation that maps percepts to actions is specifically that of automated reasoning, and the performance of all actions is the result of a conclusion reached by inferences which are, in each and every case, formally verified (which means that in the case of actions carried out by our logicist artificial agents in the coming trio of case studies, correctness is invariably proved).^6^

Next, and importantly, we point out that **Dir** is not just randomly pulled from thin air: we follow it because not doing so at best makes rational human use of artificial agents less productive and at worst makes such use in some cases outright dangerous. This holds true not only when the artificial agents in question operate in a manner divorced from the type of AI that intimately connects to argumentation (i.e., logic-based AI, to which we are adherents and which grounds the new research we present below) but also when these agents are in fact logic-based (or logicist). We explain this now with an example of each of these two types of cases.

### 2.1 The need for argumentation in non-logicist systems for rational human use

To observe the desirable role of argumentation in an example of dangerous human use of artificial agents engineered in the absence of logicist formalisms and techniques, we can consider the logic-less “large language model” Galactica, engineered and released by its creators in order to provide human beings with “a new interface for science” (Taylor et al., 2021), at least seemingly a rather laudable goal for human-centric AI.^7^ As a matter of fact, Galactica, with minimal prompts from a human, can quickly write entire scientific papers, replete with references. It does this by way of deep learning only. Unfortunately, when used by some human scientists, Galactica simply concocted many things having no relation to relevant reality. For instance, some of the references in scientific papers it “wrote” were completely fictional but of course sounded quite legitimate. The debacle, efficiently chronicled in the study mentioned in Heaven (2022), shows that Galactica poses the danger of unethical submission of scientific papers that appear sound yet are anything but. In short, a Galactic-written paper is—to use the adjective we flesh out in the study mentioned in Section 8—sophistic.

What is the solution? The solution is that the actions taken by artificial agents, in this case the assembling of scientific papers on the basis of purely statistical processing of historical data, be intimately tied to checkable arguments in support of what is expressed in said papers. As we explain below, in our argument-based AI, all outputs are the result of automatically found proofs and/or formal arguments; and these proofs and arguments can not only be inspected by humans but can be *certified* by artificial agents that automatically check these proofs/arguments.

### 2.2 The need for argumentation in logicist systems for rational human use

What about artificial agents in the second kind of case? That is, what about artificial agents that are in fact logic-based, but argumentation does not meditate between the humans using such agents and the agents' actions? An illuminating example to consider here is the famous “Monty Hall Problem” (MHP), which is going to be a bit of a theme in the present study, and which, following the study mentioned in Bringsjord et al. (2022b), we sum up as follows:

**The (3-door) Monty Hall Problem** (MHP_3_)

Jones has come to a game show and finds himself thereon selected to play a game on national TV with the show's suave host, Monty Hall. Jones is told correctly by Monty that hidden behind one of three closed, opaque doors facing the two of them is $1,000,000 USD, while behind each of the other two is a not-exactly-clean, obstreperous donkey whose value on the open market is charitably pegged at $1. Monty reminds Jones that this is a game and a fair one, and that if Jones ends up selecting the door with $1M behind it, all that money will indeed be his. (We can assume without loss of generality that Jones' net worth has nearly been exhausted by his expenditures in traveling to the show.) Monty also reminds Jones that he (= Monty) knows what is behind each door, fixed in place until the game ends.

Monty asks Jones to select which door he wants the contents of. Jones says, “Door 1.” Monty then says: “Hm. Okay. Part of this game is my revealing at this point what's behind one of the doors you didn't choose. So … let me show you what's behind Door 3.” Door 3 opens to reveal a cute but very — economically speaking — unsavory donkey. Monty now to Jones: “Do you want to switch to Door 2, or stay with Door 1? You'll get what's behind the door of your choice, and our game will end.” Monty looks briefly into the camera, directly.

What should Jones do if he is logical?

Unfortunately, as nicely explained in the study mentioned in Friedman (1998) and many other papers and books, including the recently published *Rationality* from Pinker (2021), the vast majority of humans respond by saying that Jones should not switch. In fact, as the history of MHP_3_ has shown, many mathematicians aggressively insisted that the rational policy is stay, not switch.^8^ However, the provably correct response to the question is that Jones should follow a switch policy.

Now, suppose that some artificial agents have perceived the MHP_3_ problem, automatically discovered the correct answer, and now share that answer with a typical human who fails to grasp the problem and thought that the correct policy was stay. How helpful is this artificial agent going to be to this human? Not very. After all, the human does not know *why* the correct answer is switch. The obvious solution, given the need for genuinely helpful human-centric AI, is a class of artificial agents that can not only find solutions but also provide cogent, compelling, verified arguments certifying those solutions. If such a thing is provided in the present case, the human will be enlightened. As will be observed later in the study, this is what our artificial agents can do, even in cases where the percepts to these agents are “clouded.”

## 3 The perception lacuna/challenge

The lead author has been carrying out logicist AI R&D for three decades and can count, on one hand, systems that genuinely integrate automated reasoning with the full gamut of the main human-level cognitive operators, and with attention and perception understood in keeping with state-of-the-art cognitive science. It is even harder to find such systems that are rigorous and computationally implemented. This missing type of research is what the present section's heading refers to as a lacuna.

Addressing this inadequacy is observed as important by others. For example, Dietz et al. (2022), when setting out desiderata for HCAI systems, include that such systems must exhibit a “body-mind like model of operation to sense, recognize, think, and act” (Dietz et al., 2022). For us, broadly speaking, here, “think” is constituted by reasoning,^9^ and we associate “sense, recognize” with attention and perception. Later, in the same study, when discussing what is needed for true success in HCAI and indeed for any brand of AI overall that aspires to cover the human-level case, Dietz et al. (2022) point to the following challenge:

[Such success must include showing] how the internal integrated operation of cognition, from low-level perception to increasingly higher levels of cognition, is supported by an appropriate architecture, and how an individual's cognition is integrated with the external physical and social environment (Dietz et al., 2022; emphasis ours).

In keeping with such demands, we are actively working on the integration of attention and perception with (esp. rational) human-level reasoning, in a manner that takes account of a given artificial agent's external and physical environment.^10^ Another way to put our goal of integration is to say that it is aimed at unifying “bands of cognition.” This aim is characterized by the following instructive quote:

Interestingly, [the] missing convergence toward unified theories of cognition persists across and within the bands of cognition Newell (1990). Bridging the gap between Newell's bands of cognition still exists as a problem and the main challenge remains. How do we organize the internal processes of a system at different levels such that they can operate internally linking perception and high-level cognition, by facilitating their meaningful integration with other systems and the external human participating environment? (Dietz et al., 2022).

The question in the last sentence of this quote is fundamentally what drives our integration of our automated-reasoning systems with perception; and below, Case Studies 2 (Section 7.2) and 3 (Section 7.3) demonstrate some of this study.

We now turn specifically to the latest version^11^ of our desiderata for human-level argumentation (and proof) systems, specified and implemented within the constraints of our particular approach to human-level logicist-AI.

## 4 Historical context and related work

In the present section, we first provide some historical contexts (Section 4.1) and then (Section 4.2) summarize related studies to set the stage for giving our own specific desiderata, which drive our work.

### 4.1 Historical context

Sensible presentation of our desiderata for an argument-centric automated reasoner must, at least to some degree, be contextualized historically. We, thus, now issue some remarks along this line. Needless to say, these remarks will not constitute a full history of systematic, precise work in argumentation-based formal and/or computational logic.^12^

From an historical perspective, our approach, formalisms, and AI technology for argumentation can be viewed as having roots in *dialogue logic*, the seminal 1958 introduction of which, in formal terms, is due to Lorenzen (1960). As Walton and Krabbe (1995) have pointed out, Lorenzen's work can be traced to treatment of dialogue in Aristotle (and in this connection see note 12). Since an excellent and remarkably efficient summary of dialogue logic/games is provided by Bench-Capon and Dunne (2007), a paper to which we shall return to, and rely upon, later, there is really little it makes sense for us to recapitulate from the dialogue tradition. We make only three quick points, as follows:

1. When one considers a two-person dialogue game in which Proponent and Opponent struggle over some proposition, our ShadowAdjudicator can be viewed as the judge charged with rendering rulings as to the winner.
2. We allow any number of agents to articulate and propose arguments on the proposition at hand (a fact that becomes concretized in our case studies).
3. Our third point is by far the most important of the present trio and consists of our explicitly affirming an insight into Bench-Capon and Dunne (2007), which in a word is that the specification of the internal structure of arguments, vis-à-vis conformity to inference schemata,^13^ is crucial. This insight is, in fact, explicitly included as a desideratum in **Des**, as shall be soon observed. In our case, inference schemata, as will be clearly and concretely observed in the cognitive calculi we bring to bear in our case studies, are not only formal (as is the case even with something is straightforward as *modus ponens*) but also are intensional in nature and parameter-rich (e.g., the inference schemata specified for both DCEC and IDCEC given in Section 6.2.1).^14^

Turning now to more “classical” logicist work in 20th-century AI, we begin by rehearsing that, as the reader will likely recall, standard first-order logic L_!_ (and all its fragments, such as the propositional calculus and zero-order logic L_0_^15^) is *monotonic*: the arrival of new information cannot change the result of previous inferences. That is,


$$\begin{array}{l} \text{If}\Phi ⊢\phi \text{then}\Phi \cup \text{Ψ}⊢\phi , \end{array}$$

where Φ, Ψ are sets of formulae in the formal language of L_1_, and ϕ is an individual formulae in this logic; we implicitly universally quantify over these three elements. In stark contrast, defeasible reasoning is *non*-monotonic. It has long been known in AI that such reasoning is desirable when formalizing much real-world reasoning. For instance, there are the early, classic default logics of Reiter (1980), in which epistemic possibilities hold in default of information to the contrary. In general, it is desirable to be able to reason based on beliefs which could potentially be false, and to be able to retract such beliefs when new, countervailing information arrives. Our coming desiderata **Des** call for more than this. Default logic, despite having many virtues, does not satisfy **Des**; the reason, in short, is that it has no provision for intensional/modal operators corresponding to cognitive verbs known to stand at the heart of human-level cognition (such as *believes, knows, perceives*, and *communicates*), as cognitive psychologists have shown (for an overview, see Ashcraft and Radvansky, 2013). These verbs are also known as *propositional attitudes* by logicians and philosophers, and their inclusion in a given logic makes that logic an *intensional* one, not just an *extensional* one (Fitting, 2015; Nelson, 2015).

A diagnosis parallel to that issued for default logic holds with respect to circumscription, an impressive non-monotonic form of reasoning introduced long ago by McCarthy (1980). Circumscription makes no provision for modal operators to capture cognitive attitudes and does not include the type of human-digestible arguments we require. There have been defeasible-reasoning models and systems that do include arguments that compete against each other in a manner at least somewhat similar to our concept of adjudication. The closest case in point is the work of Pollock (1995). For an excellent survey of defeasible reasoning systems that are, at least to some degree, argument-based (see Prakken and Vreeswijk, 2001).^16^

### 4.2 Related work

Argumentation in AI, as our foregoing coverage in the present section clearly indicates, is long established. To now further set the stage for enumeration of the desiderata that govern our own work, we turn to the 21st century. A truly excellent overview of this more recent work is provided by Bench-Capon and Dunne (2007), a study we have already relied upon, and which at its outset attempts to distinguish between mathematical reasoning and proofs vs. reasoning observed in arguments. The distinction is given, in part, to provide a basis for a number of studies in a special issue of *Artificial Intelligence* that follow this study, and as far as we can determine from reading these other studies, the distinction is affirmed by all the authors. However, while we certainly acknowledge that this foundational distinction is widely affirmed, it is not one that applies to our approach. In a word, the reason is that inductive logic, computationally treated, as has been explained by the lead author elsewhere (see Bringsjord et al., 2021, 2023b), must conform to the Leibnizian dream of a “universal logic” that would serve to place rigorous argumentation (in e.g., even jurisprudence) in the same machine-verifiable category as mathematical reasoning. This means that the fundamental distinction made in the study mentioned in Bench-Capon and Dunne (2007), while nearly universally accepted, does not apply to the approach taken herein. In particular, our desideratum *d*_5_ given in the next section treats proof and argument the same in this regard: both are formally, mechanically verifiable. We now take a closer look at these matters.^17^

Bench-Capon and Dunne (2007) present four properties that mathematical reasoning is said to have, but which argumentation is said to lack. We do not think that any of these properties hold of mathematical reasoning but not of argumentation; however, unsurprisingly, full analysis is beyond the present scope. We thus comment on only their fourth property, which relates directly to the issue we have already raised. This fourth property is expressed verbatim by these two authors as follows:

[I]n mathematical reasoning … [r]easoning and conclusions are entirely *objective*, not susceptible to *rational* dispute on the basis of subjective views and prejudices. Proof is demonstration whereas argument is persuasion (Bench-Capon and Dunne, 2007, p. 620).

Our reaction is rooted in Leibniz, whose objective was explicitly to do away with mere persuasion (on weighty matters), and rational disputes were to be crisply adjudicated by computation over arguments—computation we formalize and implement as automated adjudication in our sense, displayed in the present study.^18^ To meet this objective, two things were needed, a universal formal/logical language, the *characteristic universalis*, and automated-reasoning technology, the *calculus rationcinator* (Paleo, 2016). The idea is that when these are obtained, rigorous argumentation (arising from disagreements that drive the production of competing arguments) can be computationaly adjudicated, and arguments can also be discovered by computation. It is not important here at all as to whether Bringsjord and Govindarajulu have in fact found, as they claim, these two things (e.g., claimed and justified by an argument, in Bringsjord et al., 2023b); the important point is that the paradigm advanced by the research and engineering, reported herein, is based on a premeditated conflation of argument/argumentation and proof/mathematical reasoning.^19^

A second wide-ranging treatment of reasoning in AI approached via logic is provided in the study mentioned in Davis (2017), and we now offer analysis of it in relation to our own approach as well. Davis (2017) provides a survey of the attempt to formalize commonsense reasoning in a logic, and certainly some (including a reviewer of an earlier draft of the present study who encouraged us to factor in Davis's study) regard our argumentation-focused work in human-centric AI to be at least in large measure devoted to commonsense reasoning. It seems reasonable, for example, to view MHP as a commonsense-reasoning challenge. At any rate, for the sake of argument, we are more than willing to agree that this is the case. However, while the survey in question is as far as it goes in our opinion masterful, our approach is quite different in important, enlightening ways, as we now explain. We list three ways our work in computational inductive logics for formalization and automation of argumentation differs from all the work that Davis (2017) surveys:

1. *Our foundation is decidedly not mathematical logic*. Repeatedly, Davis writes that the approach he is analyzing and summarizing is the use of “mathematical logic” for formalizing commonsense reasoning. For example, on p. 651 he writes: “One of the most studied approaches toward [the] goal [of formalizing commonsense reasoning] has been to use formal *mathematical logic*” (emphasis ours). On p. 656 he writes: “This paper focuses on developing representations of fundamental commonsense domain by hand by experts using *mathematical logic* as a framework” (emphasis ours). There are other such quotes available in the study, but we omit them as redundant. The point, here, is that mathematical logic is the branch of logic devoted to formalizing mathematical reasoning, a pursuit that started with Aristotle (Glymour, 1992). However, our roots are in the tradition of devising formal logics that can capture human-level cognition, not mathematical reasoning or anything of the sort (see Bringsjord et al., 2023c). In a word, mathematical logic has for over two millennia been purely *extensional*.
2. *We straddle formal deductive logic and formal inductive logic; the latter is not on Davis's radar screen*. The phrase “inductive logic” (nor any equivalent) does not occur in Davis (2017). Given that the work surveyed therein is avowedly aligned with mathematical logic (as we have pointed out), this is unsurprising. However, formal logic is a large discipline that—as we have shared above—includes not just deductive logic but inductive logic, and the latter is itself any enormous enterprise now. There is, for example, no mention of the Carnapian edifice of pure inductive logic (Paris and Vencovská, 2015) in the survey, and no mention of inductive logic as the part of logic that includes analogical and abductive reasoning and enumerative induction (Johnson, 2016). To his great credit, Davis does consider logics in the categories of *non-monotonic, probabilistic*, and *fuzzy* (see final paragraph of p. 664). Moreover, here, there is for sure a connection to our approach and formalisms, but one important difference is that our study makes crucial use of the concept of *likelihood*, as distinct from probability (see below).
3. *There is an expressivity canyon between what Davis is concerned with vs. our cognitive calculi (*= *our logics)*. Our cognitive calculi *start* at the level of quantified multi-modal logic and expand from there. However, when Davis reports on modal logics, his orientation is that of containment. For instance, he reports with approval that “propositional modal logics … are often both expressive enough for the purpose at hand and reasonably tractable, or at least decidable” (p. 662). However, from the standpoint of human-level cognition, our position is that modal operators are almost invariably accompanied by quantification (and in fact quite naturally to L_3_).

Now, what about work specifically in defeasible argumentation systems, with an eye to the desiderata **Des** to be laid down momentarily in the next section? We wrap up the present section by summarizing two examples of such related prior study, and distinguish them from our approach in broad strokes:

1. Modgil and Prakken (2014) have presented and made available a general, computational framework—ASPIC^+^—for structured argumentation. This impressive framework is based on two fundamental principles, the second of which is that “arguments are built with two types of inference rules: strict, or deductive rules, whose premises guarantee their conclusion, and defeasible rules, whose premises only create a presumption in favor of their conclusion” (p. 31 of Modgil and Prakken, 2014). This second principle is directly at odds with desideratum *d*_5_ in the full list **Des** given in the next section. In our approach, all non-deductive inference schemata are checked, in exactly the way that deductive inference schemata are. For instance, if some inferences are analogical in nature, as long as the schema $\frac{\Phi }{C}$ (Φ for a collection of premises in some formal language and *C* for the conclusion) for an analogical inference is correctly followed, the inference is watertight, not different than even *modus ponens*, where of course specifically we have $\frac{\phi \to \psi ,\phi }{\psi }$.^20^
2. Cerutti et al. (2017) is an overview of implementation of formal-argumentation systems. However, the overview is highly constrained by two attributes. The first is that their emphasis is on Turing-decidable reasoning problems, whereas our emphasis—as reflected in **Des** and in our case studies—is on reasoning challenges that, in the general case, are Turing-undecidable. As to the second attribute, the authors are careful to say that their study is constrained by the “basic requirement” that “conflicts” between arguments are “solved by selecting subsets of arguments,” where “none of the selected arguments attack each other.” Both of these attributes are rejected in our approach; in fact, in the coming trio of case studies (Section 7), automated processing is possible *because* of this rejection. With respect to the first of their attributes, most of the interesting parts of automated-reasoning science and technology for us only *start* with problems at the level of the *Entscheidungsproblem*; see in this regard desideratum *d*_7_. As to the second attribute, it is not true for our approach.

Now, as promised, here are our desiderata, which the reader will notice are in play when we reach our case studies.

## 5 Desiderata driving our approach

We denote the 7-fold desiderata for the capability we seek in our automatic argumentation systems by ‘**Des**'. An automated reasoner of the kind we seek must:

**Desiderata “Des”**

*d*_1_     be defeasible (and hence non-monotonic) in nature (when new information comes to light, past reasoning is retracted in favor of new reasoning with new conclusions);
*d*_2_     be able to resolve inconsistencies when appropriate and tolerate them when necessary in a manner that fully permits reasoning to continue;
*d*_3_     make use of values beyond standard bivalence and standard trivalence (e.g., beyond e.g., Kleene's, 1938 true, false, and unknown trio), specifically probabilities *and* strength factors (= cognitive likelihoods), (the latter case giving rise to multi-valued inductive logics);
*d*_4_     be argument-based, where the arguments have internal inference-to-inference structure, so that justification (and hence explanation) is available;
*d*_5_     have inference schemata (which sanction the inference-to-inference structure referred to in *d*_4_), whether deductive or inductive, that are transparent, formal, and hence machine-checkable;
*d*_6_     be able to allow automated reasoning over the cognitive verbs/operators of knowledge, belief, desire, perception, intention, communication, etc., of the humans who are to be helped by this AI;
*d*_7_     be able to allow automated reasoning that can tackle Turing-unsolvable reasoning problems, e.g., queries about probability at and even above the *Entscheidungsproblem*. We do not here assume anything like hypercomputation. The requirement, here, is that formal science and engineering be harnessed to tackle *particular instances* of the Turing-uncomputable problem of algorithmically deciding provability.

We turn now to more detailed coverage of the technical background needed to understand our approach and its application in the promised three case studies.

## 6 Formal background of our brand of logicist AI

We first provide the reader with enough background to understand our approach and its application to the three case studies.

### 6.1 AI, logicist = logic-based AI, and artificial agents

AI has become a vast field as chronicled and explained in Bringsjord and Govindarajulu (2018). Accordingly, the pursuit of computing machines that qualify as intelligent and indeed even the meaning of “intelligent” itself in some contemporary debates are defined differently by different researchers and engineers, even though all of them work under the umbrella of “AI.” Our approach is a logicist one, or—as it is sometimes said—a logic-based one. A full characterization of our approach to AI and robotics is of course beyond the reach of the present study, but we must give at least enough information to orient the reader and enable understanding of our three case studies, and we do so now. We turn first to the generic concept of an *artificial intelligent agent*, or—since, by context, it is clear that we must have intelligence, in some sense, front and center—simply *artificial agents*.

#### 6.1.1 Artificial agents/AI, generically speaking

For present purposes, we rely upon how dominant textbooks, for example Russell and Norvig (2009, 2020); Luger (2008), characterize artificial agents. Their characterization is simply that such an agent computes a function from what is perceived (*percepts*) to behavior (*actions*). All such agents are assumed to operate this way in a certain *environment*, but for present purposes, we can leave explicit consideration of this aspect of the AI landscape to the side; doing so causes no loss of generality or applicability for the work we relate herein. However, what about the nature of the function from percepts to actions? As pointed out in the course of an attempt to show that the so-called Singularity^21^ is mathematically impossible (Bringsjord, 2012), the fact is that in the dominant AI textbooks, these functions are firmly assumed to be recursive. In the present study, we affirm this assumption, but the reader should keep in mind that despite this affirmation, our AI technology can still be based on automated reasoning that is routinely applied to problems that are Turing-uncomputable *in the general case*. This is directly expressed in desideratum *d*_7_ in **Des**. After all, all automated reasoners that are specifically automated theorem provers for first-order logic confront the *Entscheidungsproblem*, first shown unsolvable by Church (Church's Theorem). Our automated reasoners routinely attempt to discover arguments and proofs in order to settle queries at levels far above Church's negative result.

#### 6.1.2 The logicist approach to AI/robotics

We can now quickly state the heart of our logicist approach to AI and cognitive robotics as follows. The artificial agents we specify and implement compute their functions (from, again, percepts to actions) via automated reasoning over a given formula Φ in some formal language L for some formal logic L. This means that what these agents perceive must ultimately be transduced into content expressed in such formulae; and it means that an action, before translated into lower-level information that can trigger/control an effector, must also be expressed as a formula. The reader will see this in action below when we show our AI used in the trio of case studies. But how, specifically, are the functions computed in the case of such agents? The answer is straightforward: These functions are computed by automated reasoning. Of course, it has long been known that computation, while often understood in procedural terms (e.g., in terms of Turing machines), is fully reducible to, and usable as, reasoning.^22^

What about cognitive robotics, specifically? This is a key question because our Case Study 2 features our cognitive robot, PERI.2 (alert readers have noticed that we have already used the adjective “cognitive”). Alternatively, the introduction of cognitive elements to a formalism is said to make that formalism *behavioral* in nature; see Camerer, 2003.) We specifically pursue cognitive robotics as defined in the study by Levesque and Lakemeyer (2007),^23^ with a slight formal tweak, and say simply that a cognitive robot is one whose macroscopic actions are a function of what the robot knows, believes, intends, and so on. As seen below, these verbs are at the heart of a *cognitive calculus*, the class of cognitively oriented logics we employ in general and in automated reasoning quite concretely. It will soon be observed that the robot PERI.2 is a cognitive robot, by the definitions just given and affirmed.

Our logicist-AI work is specifically enabled by *cognitive calculi*. Details regarding this class of logics and exactly how they are tailor-made for handling cognitive attitudes/verbs are provided in numerous publications in which such calculi are harnessed for various implementations (see Govindarajulu and Bringsjord, 2017a; Bringsjord et al., 2020b). Put with a brevity here that is sufficient, a cognitive calculus C is a pair 〈L,I〉 where L is a formal language (composed, in turn, minimally, of a formal grammar and an alphabet/symbol set), and I is a collection of inference schemata (sometimes called a *proof theory* or *argument theory*) I; in this regard, our logicist-AI work is in the tradition of proof-theoretic semantics inaugurated by Prawitz (1972) and others (and for a modern treatment, see Francez, 2015; Bringsjord et al., 2022c).

Cognitive calculi have exclusively proof-theoretic and argument-theoretic semantics; no model theory is used, no possible worlds are used.^24^ Within the present study, as explained below, dialects of the cognitive calculi DCEC (deductive) and IDCEC (inductive) will be utilized, and this is what makes success in our case studies in Section 7 possible.

We said that IDCEC is an inductive cognitive calculus. The great pioneer of modern inductive logic in any form was Rudolph Carnap. Carnap would say, and the logicians and mathematicians today who continue his particular approach in the form of what is known as *pure inductive logic* (PIL) (Paris and Vencovská, 2015) would still say, that “There is a blue cube on the table” and “There is an orange sphere on the table” should each be assigned a probability value (a real number between 0 and 1, inclusive), and this content, combined with additional probabilitized propositions, can be used in a process that dictates what should be rationally believed. Unfortunately, Carnap and his followers pay precious little attention to the “coin of the realm” in human reasoning and decision-making: arguments and argumentation. This tradition (which began long before Carnap and includes e.g., Keynes and Bayes) also runs afoul of the brute fact that humans very rarely use probabilities and the probability calculus. In our approach, to computational inductive logic for AI, inference schemata that, when instantiated in sequence, lead to arguments and proofs, are front and center. This can be observed clearly in the specifications of both of the cognitive calculi used in the present study, which we now provide (next section). Later, in the three forthcoming case studies, it is the automated discovery of arguments and proofs based on linked inferences as instantiations of these schemata that is key.

### 6.2 Cognitive calculi, in more detail

Cognitive calculi, as we have said, are members of an infinite family of highly expressive logics that, for instance, include unrestricted third-order logic, meta-logical quantification, and predication (it can be expressed not only that a property has a property but that a formulae has a property), and all this extensional machinery is intertwined with intensional operators for belief, knowledge, intention, communication, action, and the traditional alethic modalities as well. To the best of our knowledge, cognitive calculi are the most expressive logics that have been implemented and used with corresponding automated reasoners. For more on cognitive calculi, see Arkoudas and Bringsjord (2009a); Govindarajulu and Bringsjord (2017a); Govindarajulu et al. (2019); Bringsjord et al. (2020b). For the shortest account of cognitive calculi, and implementation of reasoning over declarative content therein, in which it is made clear that such calculi are exclusively proof- and argument-theoretic, see Bringsjord and Govindarajulu (2020). For an explanation of how natural-language understanding works in connection with cognitive calculi, see Bringsjord et al. (2022c). There are many more resources available, as cognitive calculi are well established at this point, but for present purposes, it suffices to economically provide the specifications of the two cognitive calculi used for modeling and simulation in the present study, and these specifications follow now.

#### 6.2.1 Specifications of cognitive calculi DCEC and IDCEC

Below is the signature of the standard dialect of DCEC. The signature contains the sorts, function signatures, and grammar of this cognitive calculus, presented in a manner that is standard and self-explanatory for the most part. As obvious, lower-case Greek letters are formulae, bolded majuscule Roman letters are intensional/modal operators (**K** for *knows*, **B** for *believes*, **I** for *intends*, etc.).

**DCEC Signature**

$$\begin{array}{l} S::=\text{Agent | ActionType | Action}⊑\text{Event | Moment | Fluent}\\ f::=\{\begin{array}{l} action\;:\text{ Agent }\times \text{ ActionType }\to \text{ Action}\\ initially\;:\text{ Fluent}\to Formula\\ holds\;:\text{ Fluent}\times \text{ Moment}\to \text{Formula}\\ happens\;:\text{ Event }\times \text{ Moment }\to \text{ Formula}\\ clipped\;:\text{Moment }\times \text{ Fluent }\times \text{ Moment }\to \text{ Formula}\\ initiates\;:\text{ Event}\times \text{ Fluent }\times \text{ Moment }\to \text{ Formula}\\ ter\min ates\;:\text{ Event }\times \text{Fluent }\times \text{ Moment }\to \text{Formula}\\ prior\;:\text{Moment}\times \text{Moment }\to \text{Formula} \end{array}\\ t::=x:S|c:S\text{ | }f(t_{1},\ldots ,t_{n})\\ \phi ::=\{\begin{array}{l} q:\text{Formula}|\neg \phi |\phi \wedge \psi |\phi \vee \psi |\forall x:\phi (x)|\exists x:\phi (x)\;\\ \text{P}(a,t,\phi )|\text{ K}(a,t,\phi )|\text{ S}(a,b,t,\phi )|\text{ S}(a,t,\phi )\\ \text{C}(t,\phi )|\text{ B}(a,t,\phi )|\text{D}(a,t,\phi )|\text{ I}(a,t,\phi )\\ \text{O}(a,t,\phi ,(\neg )happens(action(a^{*},\alpha ),t^{\prime })) \end{array} \end{array}$$

**P**erceives, **K**nows, **S**ays, **C**ommon-knowledge **B**elieves, **D**esires, **I**ntends, **O**ught-to

Next is the standard set of inference schemata for DCEC. They say that when what is above the vertical line is instantiated, that which is below can be inferred (in accordance with that instantiation); this top-bottom notation is common in descriptions of so-called *natural deduction*. The approach to logicist AI-based on cognitive calculi is not restricted in any way to “off the shelf” logics but are instead created and specified for given purposes and applications in AI. However, all cognitive calculi include standard extensional logics (one or more of L_0_, L_1_, L_2_, L_3_, and standard natural-inference schemata for these extensional logics).

**DCEC Inference Schemata**

$$\begin{array}{l} \begin{array}{c} \frac{\text{K}\left(a,t_{1},\Gamma \right),\;\Gamma ⊢\phi ,\;t_{1}\leq t_{2}}{\text{K}\left(a,t_{2},\phi \right)}\left[I_{\text{K}}\right]\frac{\text{B}\left(a,t_{1},\Gamma \right),\;\Gamma ⊢\phi ,\;t_{1}\leq t_{2}}{\text{B}\left(a,t_{2},\phi \right)}\left[I_{\text{B}}\right]\\ \frac{\;}{\text{C}\left(t,\text{P}\left(a,t,\phi \right)\to \text{K}\left(a,t,\phi \right)\right)}\left[I_{1}\right]\frac{\;}{\text{C}\left(t,\text{K}\left(a,t,\phi \right)\to \text{B}\left(a,t,\phi \right)\right)}\left[I_{2}\right]\\ \frac{\text{C}\left(t,\phi \right),\;t\leq t_{1},\ldots ,t\leq t_{n}}{\text{K}\left(a_{1},t_{1},\ldots \text{K}\left(a_{n},t_{n},\phi \right)\ldots \right)}\left[I_{3}\right]\frac{\text{K}\left(a,t,\phi \right)}{\phi }\left[I_{4}\right]\\ \frac{t_{1}\leq t_{2}\leq t_{3}}{\text{C}\left(t,\text{K}\left(a,t_{1},\phi_{1}\to \phi_{2}\right)\right)\to \text{K}\left(a,t_{2},\phi_{1}\right)\to \text{K}\left(a,t_{3},\phi_{2}\right)}\left[I_{5}\right]\\ \frac{t_{1}\leq t_{2}\leq t_{3}}{\text{C}\left(t,\text{B}\left(a,t_{1},\phi_{1}\to \phi_{2}\right)\right)\to \text{B}\left(a,t_{2},\phi_{1}\right)\to \text{B}\left(a,t_{3},\phi_{2}\right)}\left[I_{6}\right]\\ \frac{t_{1}\leq t_{2}\leq t_{3}}{\text{C}\left(t,\text{C}\left(t_{1},\phi_{1}\to \phi_{2}\right)\right)\to \text{C}\left(t_{2},\phi_{1}\right)\to \text{C}\left(t_{3},\phi_{2}\right)}\left[I_{7}\right]\\ \frac{\;}{\text{C}\left(t,\forall x.\;\phi \to \phi \left[x\mapsto t\right]\right)}\left[I_{8}\right]\frac{\;}{\text{C}\left(t,\phi_{1}↔\phi_{2}\to \neg \phi_{2}\to \neg \phi_{1}\right)}\left[I_{9}\right]\\ \frac{\;}{\text{C}\left(t,\left[\phi_{1}\wedge \ldots \wedge \phi_{n}\to \phi \right]\to \left[\phi_{1}\to \ldots \to \phi_{n}\to \phi \right]\right)}\left[I_{10}\right]\\ \frac{\text{B}\left(a,t,\phi \right)\;\text{B}\left(a,t,\phi \to \psi \right)}{\text{B}\left(a,t,\psi \right)}\left[I_{11a}\right]\;\frac{\text{B}\left(a,t,\phi \right)\;\text{B}\left(a,t,\psi \right)}{\text{B}\left(a,t,\phi \wedge \psi \right)}\left[I_{11b}\right]\\ \frac{\text{S}\left(s,h,t,\phi \right)}{\text{B}\left(h,t,\text{B}\left(s,t,\phi \right)\right)}\left[I_{12}\right]\frac{\text{I}\left(a,t,\text{happens}\left(\text{action}\left(a^{*},\alpha \right),t^{\prime }\right)\right)}{\text{P}\left(a,t,\text{happens}\left(\text{action}\left(a^{*},\alpha \right),t^{\prime }\right)\right)}\left[I_{13}\right]\\ \frac{\begin{array}{cc} \text{B}\left(a,t,\phi \right) & \;\text{B}\left(a,t,\text{O}\left(a,t,\phi ,\chi \right)\right)\;\text{O}\left(a,t,\phi ,\chi \right) \end{array}}{\text{K}\left(a,t,\text{I}\left(a,t,\chi \right)\right)}\left[I_{14}\right] \end{array} \end{array}$$

The following two framed boxes specify the additional signature and inference schemata for IDCEC, respectively. That is, they build on top of those given for DCEC immediately above. These specifications enable reasoning about uncertain belief. In the first of three case studies discussed next, we will describe the uncertainty system which enables the ascription of *likelihood* values to beliefs present in these schemata. Herein, we only provide a subset of the inference schemata of IDCEC; a full exposition of IDCEC and its inference schemata are the focus of a doctoral dissertation (Giancola, 2023). For an early inductive cognitive calculus with cognitive likelihood, see Govindarajulu and Bringsjord (2017b).

**Additional Signature for IDCEC**

$$\begin{array}{l} S::=\text{Number | List}\\ f::=\{\begin{array}{l} \min\text{ : List}[\text{Number}]\;\to \text{Number}\\ \max\text{ : List}[\text{Number}]\;\to \text{Number} \end{array}\\ \phi ::=\{{\text{B}}^{\sigma }(a,t,\phi )\\ \text{where }\sigma \in [-5,-4,\ldots ,4,5] \end{array}$$

**Additional Inference Schemata for IDCEC**

$$\begin{array}{l} \frac{\text{S}\left(s,a,t_{1},\phi \right),\;t_{1}<t_{2}}{{\text{B}}^{1}\left(a,t_{2},\phi \right)}\left[I_{1}^{\ell }\right]\frac{\text{P}\left(a,t,\phi \right)}{{\text{B}}^{4}\left(a,t,\phi \right)}\left[I_{4}^{\ell }\right]\\ \frac{{\text{B}}^{\sigma }\left(a,t_{1},\phi \right),\;\Gamma ⊬\neg {\text{B}}^{\sigma }\left(a,t_{2},\phi \right),\;t_{1}<t_{2}}{{\text{B}}^{\sigma }\left(a,t_{2},\phi \right)}\left[I_{\text{PROP}}^{\ell }\right]\\ \frac{{\text{B}}^{\sigma_{1}}\left(a,t,\phi_{1}\right),\ldots ,{\text{B}}^{\sigma_{m}}\left(a,t,\phi_{m}\right),\;\left\{\phi_{1},\ldots ,\phi_{m}\right\}⊢\phi ,\;\left\{\phi_{1},\ldots ,\phi_{m}\right\}⊬⊥}{{\text{B}}^{min\left(\sigma_{1},\ldots ,\sigma_{m}\right)}\left(a,t,\phi \right)}\left[I_{\text{WLP}}^{\ell }\right]\\ \text{where}\sigma_{i}\in \left[0,1,\ldots ,4,5\right] \end{array}$$

#### 6.2.2 Regarding metatheoretical properties of our cognitive calculi and associated automated reasoners

As the chief purpose of the study we report herein is to advance logicist AI, both formally and computationally, rather than to advance computational formal logic in and of itself, it would be inappropriate to spend appreciable time and space explaining, let alone proving, the metatheoretical properties—soundness, completeness, un/decidability, complexity measures, etc.–of the family of cognitive calculi and the members thereof used herein (DCEC& IDCEC) and of our automated reasoners. However, we do now provide some brief metatheoretical information that readers well versed in formal logic will likely find helpful.

To begin, recall that desideratum *d*_7_, if satisfied, ensures that the fundamental question as to whether some formula ϕ can be inferred (*via* some collection of inference schemata) from some set Φ of formulae is for us usually^25^ Turing-undecidable. We have already mentioned Church's Theorem in this regard, which of course applied to theoremhood in first-order logic = L_1_. However, as a matter of fact, L_1_ is *semi-decidable*: if, in fact, there exists a proof in the first-order case that supports an affirmative answer to the question, that proof can be algorithmically found. However, in the case of our paradigm, there are many general inference questions posable by and to our artificial agents using as a basis a cognitive calculus (whether deductive or inductive) that are fully undecidable. This can be immediately observed from the well-known theorem that L_2_ is not even semi-decidable.^26^ However, our study, as it is based on cognitive calculi, places crucial reliance upon human-level cognitive verbs, where these verbs are logicized by relevant modal operators; for example: **P** for *perceives*, **B** for *believes* (which, in our approach, can have a positive likelihood parameter attached), **K** for *knows* (which also can have a positive likelihood parameter attached), and so on. This means that things are only that much harder computationally, and in fact, since both the Arithmetic and Analytic Hierarchies are purely extensional (the former based on L_1_ and the latter based on L_2_), and hence devoid of modal operators, things are only even harder, given our willingness to consider formulae and queries arising from an unflinching look at the human case. This is simply the nature of the beast—that beast being the undeniable expressivity of human-level cognition and specifically of human-level argumentation. After all, there can be no denying that humans create and assess arguments that, when logicized, require remarkably high levels of expressivity; this holds for even everyday activity, not just for recherché academic problems. For an everyday example, let us consider an argument, to be found and verified by our AI technology, for the proposition (‡) that the dog Rover is scary, based chiefly on these two premises:

(P1) As trainer David knows, there are some properties that are downright scary and that some dogs have; and if they have any of these properties, the dog in question is itself scary.
(P2) David also knows that one of these scary properties is having prominent and pronounced musculature, and another is having long and large incisors.

Now further suppose that (P3) David perceives a particular dog, Rover, who as it happens has thick, pronounced incisors and prominent pronounced musculature. Our automated reasoner, ShadowProver, working with the formal representation of {P1, P2, P3} in the cognitive calculus DCEC^3^,^27^ is able to find an argument, and verify it, for (‡)—despite the formal fact that, in the general case, the question as to whether a proposition follows from modalized third-order formulae is a Turing-undecidable question.^28^

Some readers, even cognoscenti, may then ask: But if the queries your artificial agents much seek to handle are this difficult, how does the engineering of your automated-reasoning systems work? This question alone, if answered fully, would require its own monograph. However, the answer is actually quite simple, fundamentally, The short version of the answer is that our engineering (a) reflects the famous conception, originated by AI pioneer Herbert Simon, of “satisficing” (Simon, 1956); and (b) this engineering makes use of a most valuable but low-technology sub-system: a stopwatch, in the form of timeouts on duration of CPU processing. In other words, we engineer for success on particular cases within the general space of Turing-uncomputable problems, and if processing takes too long and no answer has been returned, we curtail processing by fiat, in accordance with a pre-set length of time allowed for CPU activity. In the case of our three case studies featured herein, temporal thresholds were not reached, in fact were not even approached.^29^

What about other metatheoretical properties in the realm of formal logic? What about complexity, soundness, completeness, for example? Complexity is irrelevant, because almost all of the problems that our human- and argumentation-centric artificial agents seek to solve are not even in the Polynomial Hierarchy (since they are above Σ_1_ in the Arithmetic Hierarchy). Soundness and completeness, given that our approach is purely proof-theoretic, is beyond scope; readers for a start are directed to Govindarajulu et al. (2019). As can be readily understood given the foregoing, while there is a lot of truly impressive work in AI and intelligent systems that makes use of computational logic, much of it is nonetheless radically different in formal orientation than ours. An example is the use of logic programming. For a specific example, as Brewka et al. (2011) show, *answer set programming* (ASP) is quite powerful and promising—but its nature is applauded and affirmed because “ASP … aim[s] to maintain a balance between expressivity, ease of use, and computational effectiveness” (Brewka et al., 2011, p. 92–93). The balance, here, can indeed be very powerful, but as should be abundantly clear, our approach and the concrete case studies within it reported herein, we do not desire this balance.^30^

One final word, aimed especially at those who subscribe, as the first author long did but no longer does, to the general expressivity-vs.-tractability tradeoff for formal (extensional) logics that has become part of the fixed furniture of logicist AI. This tradeoff, entrenched since at least the publication of the important (Levesque and Brachman, 1985), is far from being both clear and ironclad in the case of our brand of AI engineering. The logico-mathematical reason stems directly from Gödel's Speedup Theorem (GST) (Buss, 1994, 1995), which, in word, says that the move from first- to-second-order logic enables a non-recursive gain in efficiency, measured by length of proof (and likewise for jumping from second- to-third-order, and so on for each jump).^31^ In engineering terms, while of course we have no recourse to algorithms for answering queries fully in the general case, we also know that engineering techniques just might find staggering gains in efficiency for cases at hand. Readers interested in learning more about this phenomenon are advised to start with the striking example of Boolos (1987) and move from there to study GST itself via the references we provided.

## 7 Three Case Studies

We turn now to our three case studies. In the third and final study, reasoning is explained in somewhat higher-level terms than in the case of the first and second; more specifically, the arguments in Case Study 3 are for space-saving and expository purposes expressed rather informally. Our first study takes us back to Monty Hall, and we proceed to it now.

### 7.1 Case Study 1: MHP_3_ redux

We have every confidence the reader will remember MHP_3_, which we suppose that some artificial agents have perceived in full, automatically discovered the correct answer for, and now share that answer with a typical human who fails to grasp the problem, and thought the correct answer was stay. How helpful is this artificial agent going to be to this human? Not very. After all, our human does not know *why* the correct answer is switch. The obvious solution, given the need for genuinely helpful human-centric AI, is a class of artificial agents that can not only find solutions but also provide cogent, compelling, verified arguments, certifying those solutions. If such a thing is provided in the present case, the human will be enlightened. This is what our artificial agents can do.

Given the complexity of MHP_3_, we cannot, herein, canvass the full terrain of this problem, its logicization into our inductive logic IDCEC, and solutions automatically found, but let us consider two prominent arguments regarding MHP_3_, the first sound (and hence both veracious and valid^32^) and the second not. The sound argument goes as follows:

1. Without loss of generality, assume that you select Door 1.^33^
2. There are three potential cases, in which the prize is behind Door 1, Door 2, or Door 3, respectively.
3. Let's first consider the outcome of the three cases under the stay protocol.
  (a) If the prize is behind Door 1, you win. If it is behind Door 2 or 3, you lose.
  (b) Hence there is a $\frac{1}{3}$ chance of winning if you follow stay.
4. The cases are a bit more complex if you follow switch, because, crucially, Monty *knows* where the prize is, and, having *perceived* your initial choice, will *always* reveal a door without the prize behind it.
  (a) If the prize is behind Door 1, you will lose. Monty can open either of Door 2 or Door 3 (and should be assumed to randomly choose which one), and regardless of which door you switch to, you will lose.
  (b) If the prize is behind Door 2, Monty *must* open Door 3. Therefore if you switch to Door 2, you will win.
  (c) If the prize is behind Door 3, Monty *must* open Door 2. Therefore, if you follow switch and move to Door 2, you will win.
  (d) Hence, by simply counting, we deduce that there is a $\frac{2}{3}$ chance of winning if you follow switch.

While many arguments have been made for stay,^34^ they mostly follow the same general pattern. That pattern is as follows:

1. Without loss of generality, assume that you select Door 1, and that Monty then opens Door 3.
2. When Monty opens Door 3 that door of course has dropped out of consideration, and we are down to two doors, so the probability that the prize is behind Door 1 becomes ½ same as the probability that the prize is behind Door 2.
3. Hence there is no reason to switch doors (and since—as the economists who study rationality say—time is money, switching is irrational).

Pinpointing where this invalid argument goes awry is enabled by our concept of *likelihood*, specifically what we term *cognitive* likelihood (Giancola, 2023). The invention of this concept and its use in our intelligent, defeasible argumentation systems satisfies desideratum *d*_3_. This concept enables the ranking of the strength of beliefs (and other cognitive attitudes), in accordance with their likelihood values. The spectrum of the 11 possible values are presented in Table 1 (the caption for which offers some contextualization of these values in contrast with probabilities). The use of these strength-factor/cognitive likelihood values makes IDCEC a multi-valued (or many-valued) logic; an efficient, broad overview of such logics is provided in the study by Gottwald (2015).^35^

**Table 1:** The 11 cognitive likelihood values.

**Numerical**	**Linguistic**
5	certain
4	evident
3	overwhelmingly likely
	= beyond reasonable doubt
2	likely
1	more likely than not
0	counterbalanced
-1	more unlikely than not
-2	unlikely
-3	overwhelmingly unlikely
	= beyond reasonable belief
-4	evidently not
-5	certainly not

These values, notably, are not in any way real numbers in an interval, as are probabilities in Kolmogorov's (1933) probability calculus (the interval of course being [0, 1]), much used in modern AI, e.g., in Bayesian approaches. Rather, these are fixed values in the traditional sense of ‘value' in multi-valued (or many-valued) logics, where each value has an independent justification as a determinate value in rational human cognition. For example, when strength/value is 3 for a belief, this corresponds to what humans in general refer to as something that ought (epistemically, not morally, speaking) to be believed because the proposition is “beyond reasonable doubt,” a concept central to occidental jurisprudence. For the present study, it is beyond scope to present our full axiomatic theory L of cognitive likelihood that is subsumed by IDCEC, in which Kolmogorov's axioms do not hold. E.g., where *p* yields the probability of an event/proposition ϕ, Kolmogorov's second axiom says that if ϕ is a theorem in a standard, elementary extensional logic (such as the propositional calculus), *p*(ϕ)=1. However, theorems in such a logic are not at all guaranteed to have a likelihood value of 5, since an infinite number of such theorems are not familiar to human beings and hence cannot be believed. In addition, theorems of L are often completely without corresponding analogs in the probability calculus. E.g., “if ℓ(ϕ) = 5, ℓ(¬ϕ) = 0” is a theorem in L that has no analog in the probability calculus.

By enabling beliefs to take on these uncertainty levels, cognitive likelihood allows agents to reason with uncertain beliefs generated by and reasoned over in integration with other modalities, for example, with perception, communication, and intention. This is formalized in the inference schemata of IDCEC. For example, perception of ϕ sanctions, by inference schema $I_{4}^{\ell }$ (see the specification of inference schemata in the specifications shown in Section 6.2.1), a belief that ϕ—but only at the cognitive-likelihood value σ: = 4. (that which we perceive, at least when we are talking about perception of things in the external world, might be illusory). Certainty, when σ: = 5, is reserved in our framework for belief regarding mathematical propositions. In general, this ability to reason with cognitive-likelihood values enables the kind of nuanced argumentation we seek, as it provides a formalism in which individual statements and arguments as a whole can be assigned relative strengths (= cognitive likelihoods), which, in turn, allows certain statements and arguments carrying higher strength to “defeat” others non-monotonically as time flows; this occurs in our case studies.

Now, back to MHP_3_. The first argument is fully supported by the basic tenets of probability theory viewed through the lens of odds (i.e., the probability of an event is the ratio of the number of possible outcomes in which it occurs, over the number of total possible outcomes).^36^ Therefore, a belief in the conclusion of Argument 1—namely, that one should follow switch—can be held at the level of evident. It is evident, not certain, because the argument fundamentally relies on the agent's perception of various elements of the game, which could be compromised without violation of any mathematically necessary axioms or theorems. Such beliefs are inferred using schema $I_{4}^{\ell }$ as follows:


(1) $$\begin{array}{l} \frac{\text{P}\left(a,t,\phi \right)}{{\text{B}}^{4}\left(a,t,\phi \right)}\left[I_{4}^{\ell }\right] \end{array}$$

On the other hand, Step 2. of Argument 2 is generally asserted with no justification. One could argue that it is justified by the large group of people who state it. Given the inference schema $\left[I_{2}^{\ell }\right]$, such a justification can warrant a belief at the level of more likely than not but not higher. Therefore, we have formally observed that the first argument is stronger than the other and hence should be accepted.

**Figure 1:**
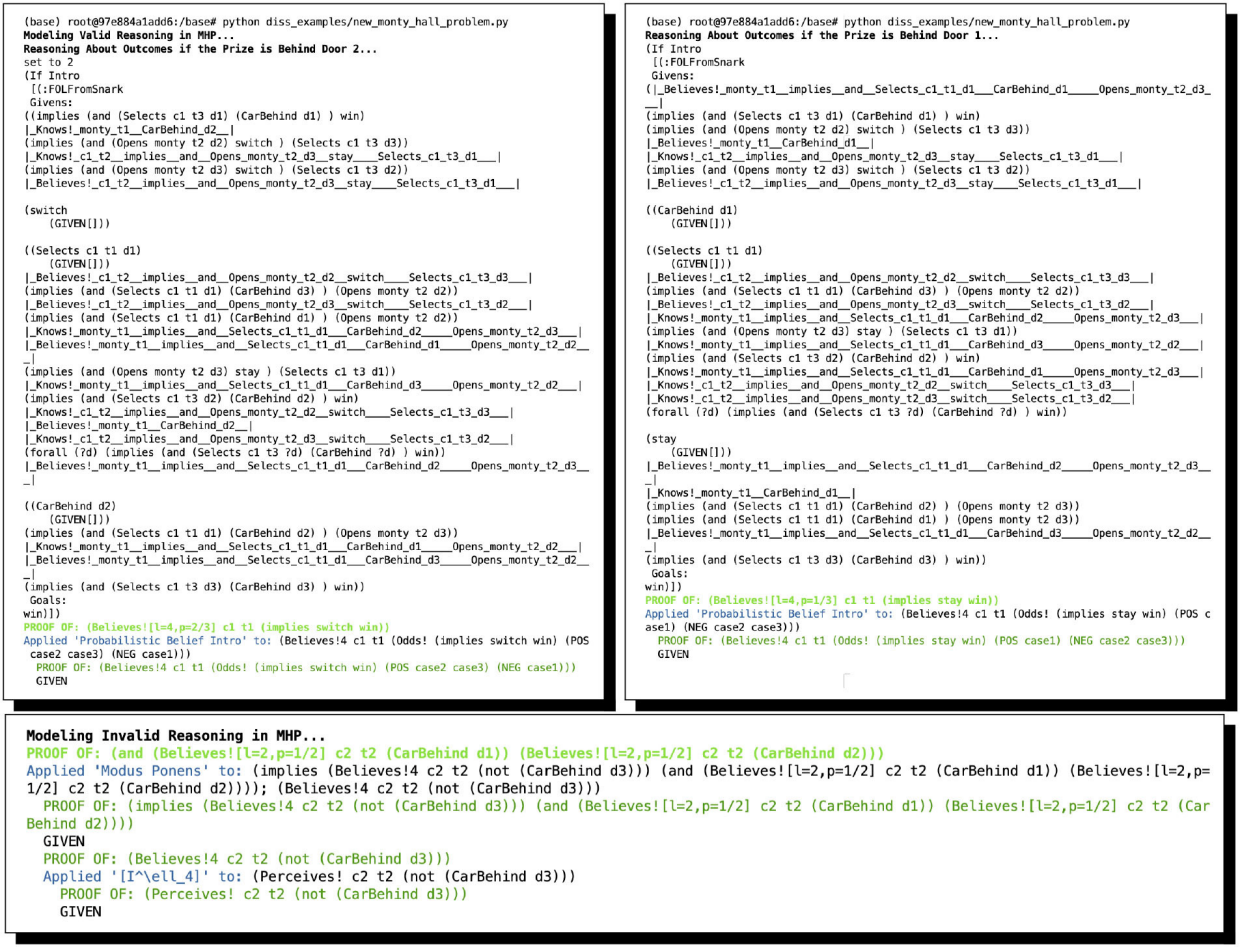
Two arguments for supposedly solving MHP_3_, automatically found by ShadowAdjudicator/ShadowProver. The complete valid argument includes six sub-proofs, the result of considering whether switching or staying will result in a win depending on the three possible locations of the prize (and assuming, without loss of generality, that the contestant initially selected Door 1). In the graphic here, we show two of the six: switching when the prize is behind Door 2, and staying when the prize is behind Door 1. One of the others is the same as one shown: the contestant wins if they switch when the prize is behind Doors 2 or 3. The other 3 proofs result in failure; e.g., one cannot prove that staying will result in a win if the prize is behind Doors 2 or 3.

As mentioned above, while a full formal and computational account of the overarching argument and its sub-proofs are out of scope in the present study, we give the automated proofs found by ShadowAdjudicator in Figure 1 and point the interested reader to Giancola (2023) for a full exposition of the relevant inference schemata, all the arguments and proofs, and full analysis. We mention as well that there are now numerous variants of MHP_3_ that are a good deal trickier than the original; these are comprehensively treated in the study by Bringsjord et al. (2022b), which takes account, for instance, of the variants discussed in the study by Rosenthal (2008).

### 7.2 Case Study 2: the robot PERI.2 meets “Clouded” Meta-Forms

**Figure 2:**
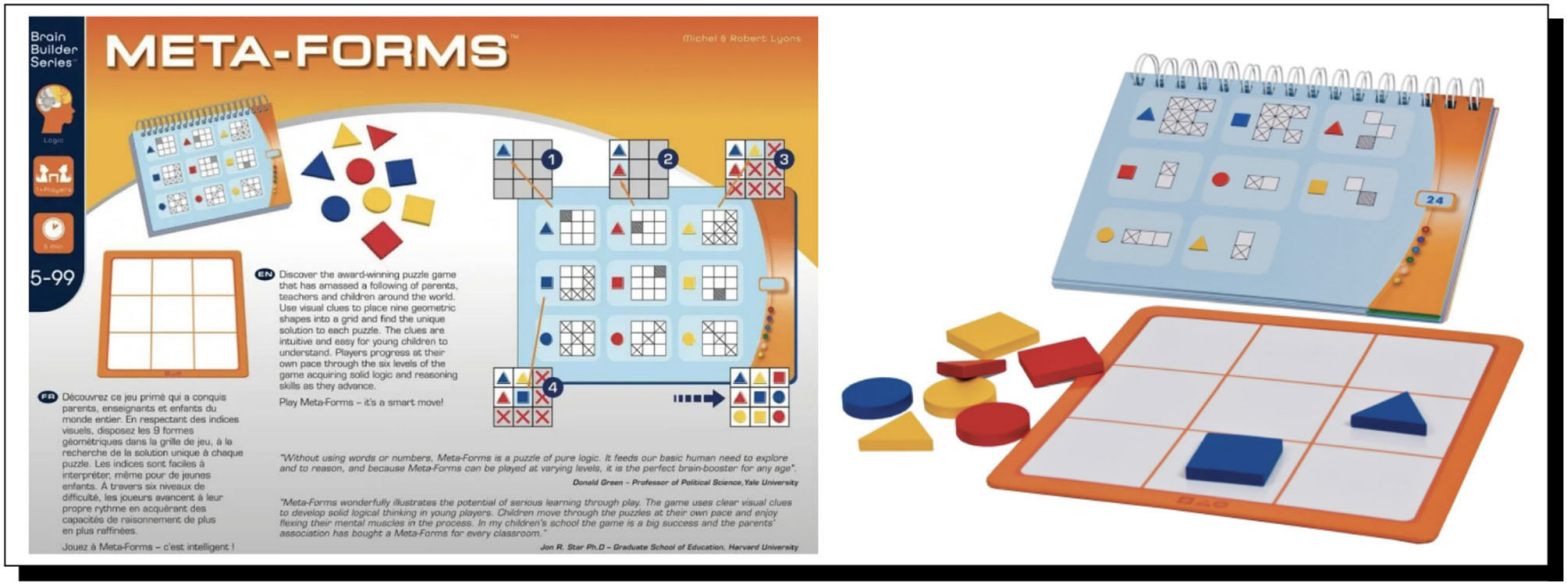
The Meta-Forms game, from FoxMind. This game provides a series of “clues” to the would-be puzzle solver, each of which is a visual version of a “logical statement,” which, in turn for our AI work, becomes a formula in a cognitive calculus (often requiring for such logicization only the formal language of a standard extensional logic such as L_1_). The goal is to physically construct a complete configuration of the 3 × 3 board from these clues, i.e., a full placement of each of the nine different objects in the game (3D versions of a triangle, square, and circle, each of which can be one of the three colors of red, blue, and yellow). Formally, if Π is a complete configuration of the board, and Γ the collection of formulae that logicize all clues, necessarily Π∪Γ is provably consistent in L_1_ and more expressive logics that subsume it.

Our second case study revolves around a very interesting and challenging reasoning game that we are using in a sustained attempt to quite literally have the cognitive robot PERI.2^37^ attend school and progress grade-by-grade through at least high school, on the road thereby to artificial general intelligence (AGI); this project was announced in Bringsjord et al. (2022a). The game is called “Meta-Forms” (see Figure 2 for a rapid orientation to the game).

**Figure 3:**
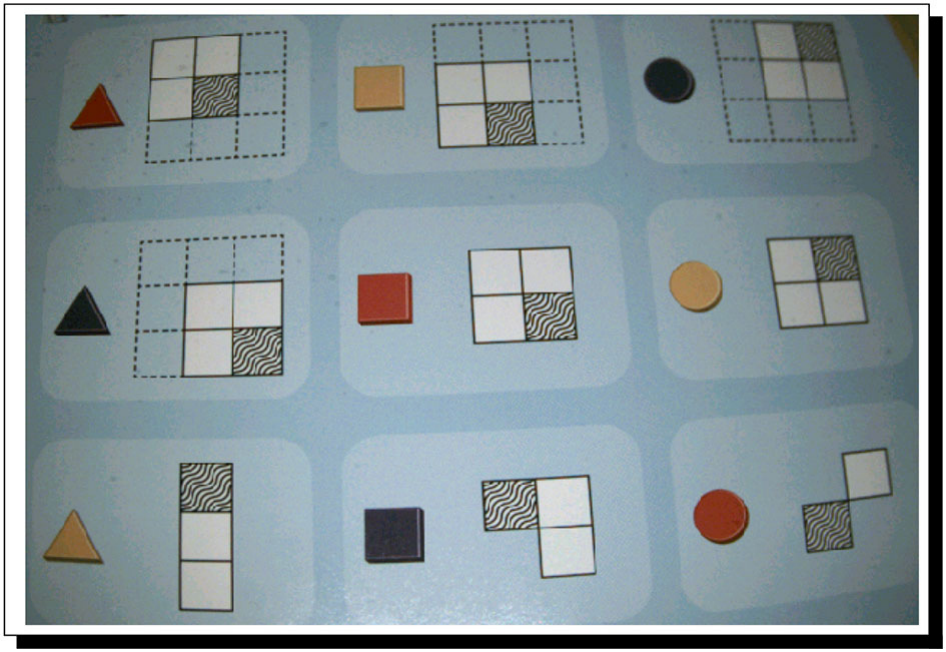
A Meta-Forms problem. This is the full problem that serves as the challenge at the heart of Case Study 2. Clues are best read by the AI system top to bottom and left to right. The goal is to reason out where to place all nine objects on the grid.

For our second case study, PERI.2 is issued the challenge of solving a Meta-Forms problem; not one of the very hardest of such problems, but certainly a non-trivial one, even for adult humans; the problem is shown in Figure 3.

**Figure 4:**
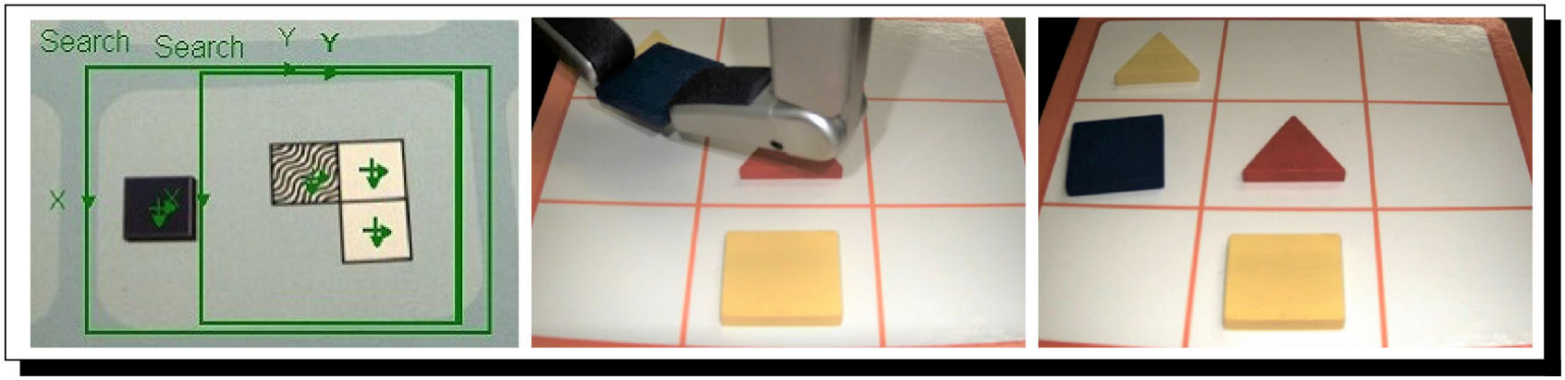
PERI.2 observes the clue **(left)** and holds a Meta-Form piece in One Hand **(center)**, correctly placing the shape **(right)**. The clue, when logicized by PERI.2, can be represented as: **B**[*peri2, now, LocatedAt*(*bluesquare*, 1)∨*LocatedAt*(*bluesquare*, 2)∨*LocatedAt*(*bluesquare*, 4) ∨*LocatedAt*(*bluesquare*, 5)]. Notably, this is a disjunction. The challenge is to dynamically adjust arguments through time as clues are perceived by trying to negate disjuncts. Machine-vision middleware for PERI.2 is courtesy of Cognex, three of whose cameras are part of PERI.2 as well; hands are from Barrett Technologies.

**Figure 5:**
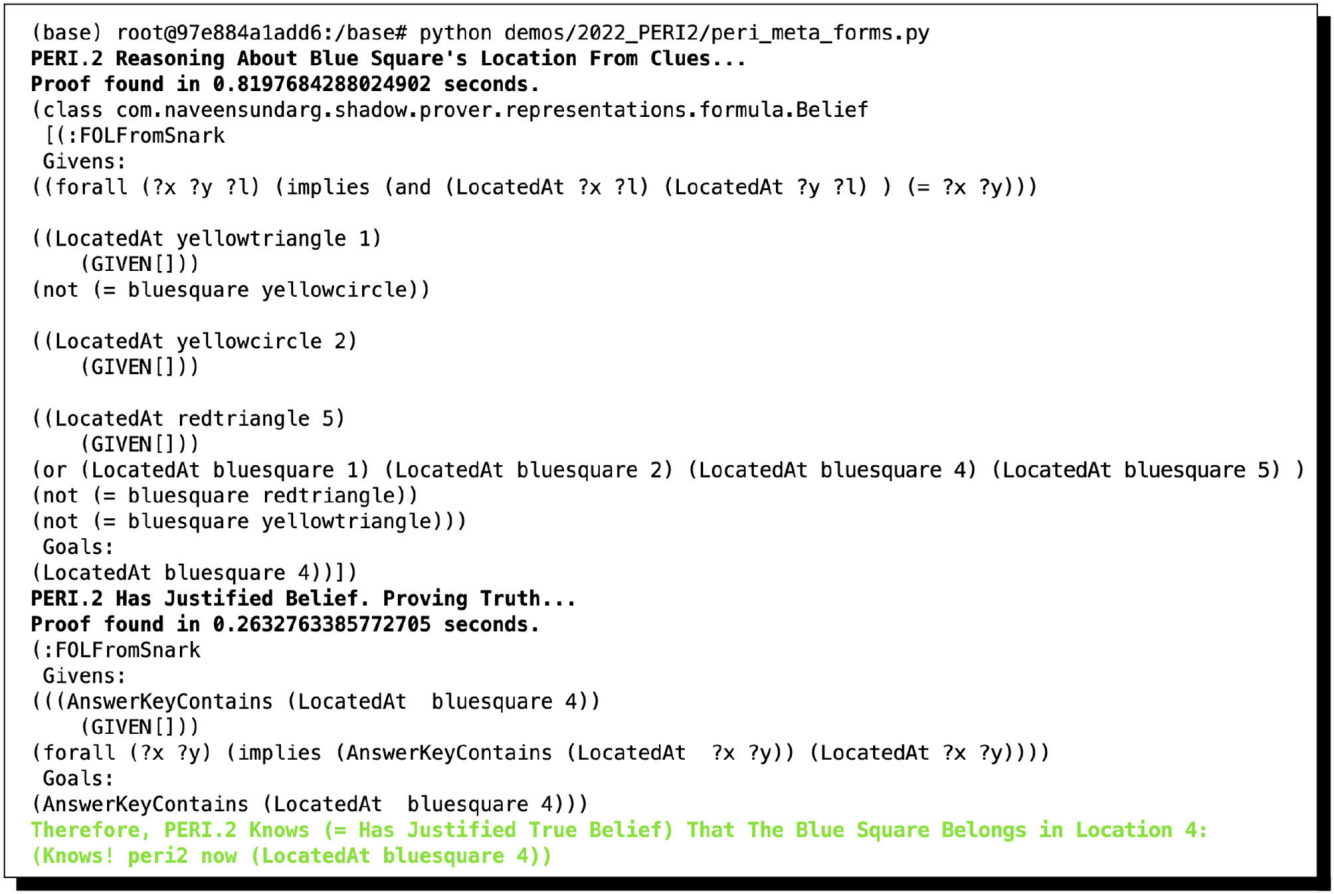
PERI.2 comes to know by reasoning that the Blue Square is at location #4. A rather long run of automated reasoning eventuates in PERI.2's coming to know that the blue square is at location #4. The proof given here provides justification for PERI.2's belief. It is, in fact, true that the blue square belongs to location 4. Therefore, in accordance with the conception of knowledge as justified true belief, where both belief and knowledge are allowed to vary in strength [in order to surmount the famous problem of Gettier (1963), as explained in Bringsjord et al. (2020b)], PERI.2 knows the correct placement.

PERI.2 does meet with success, in what as far as we know is one of the most robust uses of argumentation-based AI in cognitive robotics. This success is shown in Figure 4, and the automatically found reasoning that leads to PERI.2's knowledge^38^ (which, in turn, leads to the intention to act accordingly, and then the performance of the action) is shown in Figure 5. It is important to realize that because of the nature of Meta-Forms problems, dynamic argumentation through time is part and parcel of how PERI.2 operates.

**Figure 6:**
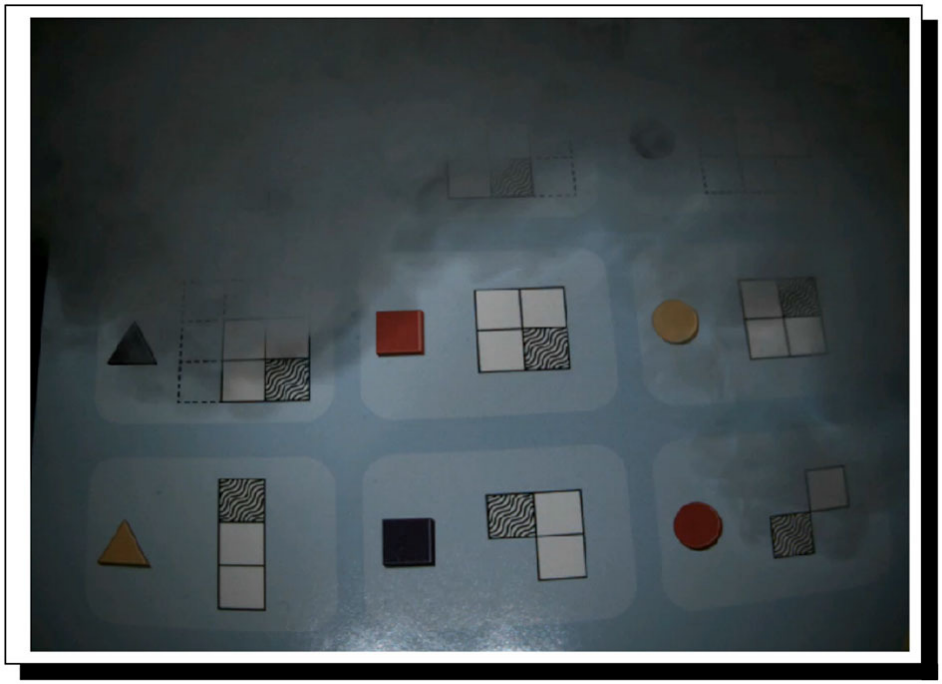
A Full Trio of clues are fogged over. Fog (courtesy of a fog machine) has appeared in the RAIR Lab, and the results are not good perception-wise.

**Figure 7:**
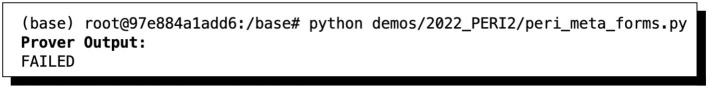
PERI.2 fails to find a proof when perception is compromised. Due to fog in the environment, some key clues are now absent in automated reasoning, and there is failure because PERI.2 cannot turn disjunctive (indeterminate) clues into knowledge.

However, now what happens if PERI.2's environment is uncoöperative? Specifically, what happens when this cognitive robot is faced with fog (or smoke, etc.), to the point where some possibly crucial information cannot be perceived, then believed, and then reasoned about? Such a situation is shown in Figure 6. In this situation, PERI.2 is unable to arrive at knowledge in support of action that can be taken in order to physically solve the problem (see Figure 7).

### 7.3 Case Study 3: a life-and-death multi-agent decision

The ARCADIA human-level cognitive architecture (Bridewell and Bello, 2015) provides means by which we are able to integrate our cognitive calculi and associated automated reasoners with a perceptual system that takes into account not only the general cognitive science of perception but also specifically a given agent's dynamically shifting attention. Computational cognitive science has disclosed that attention and perception go hand in symbiotic hand, and when an agent is designed and implemented as an ARCADIA model, this symbiosis is made computationally real.

In the present section, we give a case study of a robust multi-agent system perceiving and reasoning, and in which our automated-reasoning technology helps assess threat levels in a delicate scenario that is too depressingly real in the world today. The simulation is in real time, as perceptual information is communicated to and from multiple agents. However, before the case study, we give now some brief—but given purposes—additional relevant background on ARCADIA.

The ARCADIA cognitive architecture is composed primarily of a collection of non-introspectable processing units called *components*. On each ARCADIA processing cycle, components may take in and produce *interlingua content items*, which are tables of labeled data able to be interpreted by other components. Once generated, content items are placed in an accessible content area from which the architecture will select one on each processing cycle to become the *focus of attention*. This selected element is fed back into the components and used to generate more content items. The strategy for selecting a content item is decided on a task-to-task basis that favors items, representing things deserving of more attention, such as those representing changes to objects within the field of vision. Though this architectural design and various types of components are motivated, as we have said, by the cognitive science of cognition, ARCADIA is able to smoothly and efficiently perform a robust range of tasks as implemented computation—such as object recognition, tracking, and driving (Bello and Bridewell, 2020).

To move into the case study, let us suppose that it is known that some people of interest are working on an unknown device in a building in an area that has a history of terrorist training and planning.^39^ A team of “blue” artificial agents is tasked with deciding (and reporting to humans thereafter) whether or not these people of interest and the device with them pose a threat. The investigating team operates under the two-part assumption that those in the building are possibly terrorists, and the device in question possibly a bomb. In total, there are four investigative artificial agents. Three of them are in the vicinity of the building and are approaching it to ascertain the nature of the device in question via their sensors. These three agents are a high-altitude drone with a scanner (denoted by constant *hdrone*), a low-altitude drone with a camera (denoted by constant *ldrone*), and a land-based agent with wall-penetrating radar (denoted by constant *radar*). The final agent is a special argument-adjudicating agent (*adjudicator*) in full command of both cognitive calculi DCEC and IDCEC and also ShadowProver and ShadowAdjudicator; this agent is tasked with sending mission commands and receiving messages from the other agents. From these messages, it is to use all its information at each time step to determine by reasoning if the people and the device are a threat. The other agents do not have full cognitive power (i.e., most of the cognitive verbs captured by both DCEC and IDCEC cannot be instantiated by their processing; e.g., these agents do not have the epistemic “power” of *believing* and *knowing*); rather, they are only *perceptive* and *communicative agents*, able to focus on commands and changes in their environment and report their percepts to the adjudicator agent. The adjudicator agent is, thus, able to reason about the state of the world using the full ensemble of our calculi and automated reasoners, but the subsidiary agents are restricted to proper parts of the cognitive calculi in question. Both DCEC and IDCEC have in their formal languages both a perception operator **P** and a communication operator **S**, read as “says” (see again as needed Section 6.2.1); but the operators in this pair for belief, knowledge, intention, and action are not available to the subsidiary agents.

**Figure 8:**
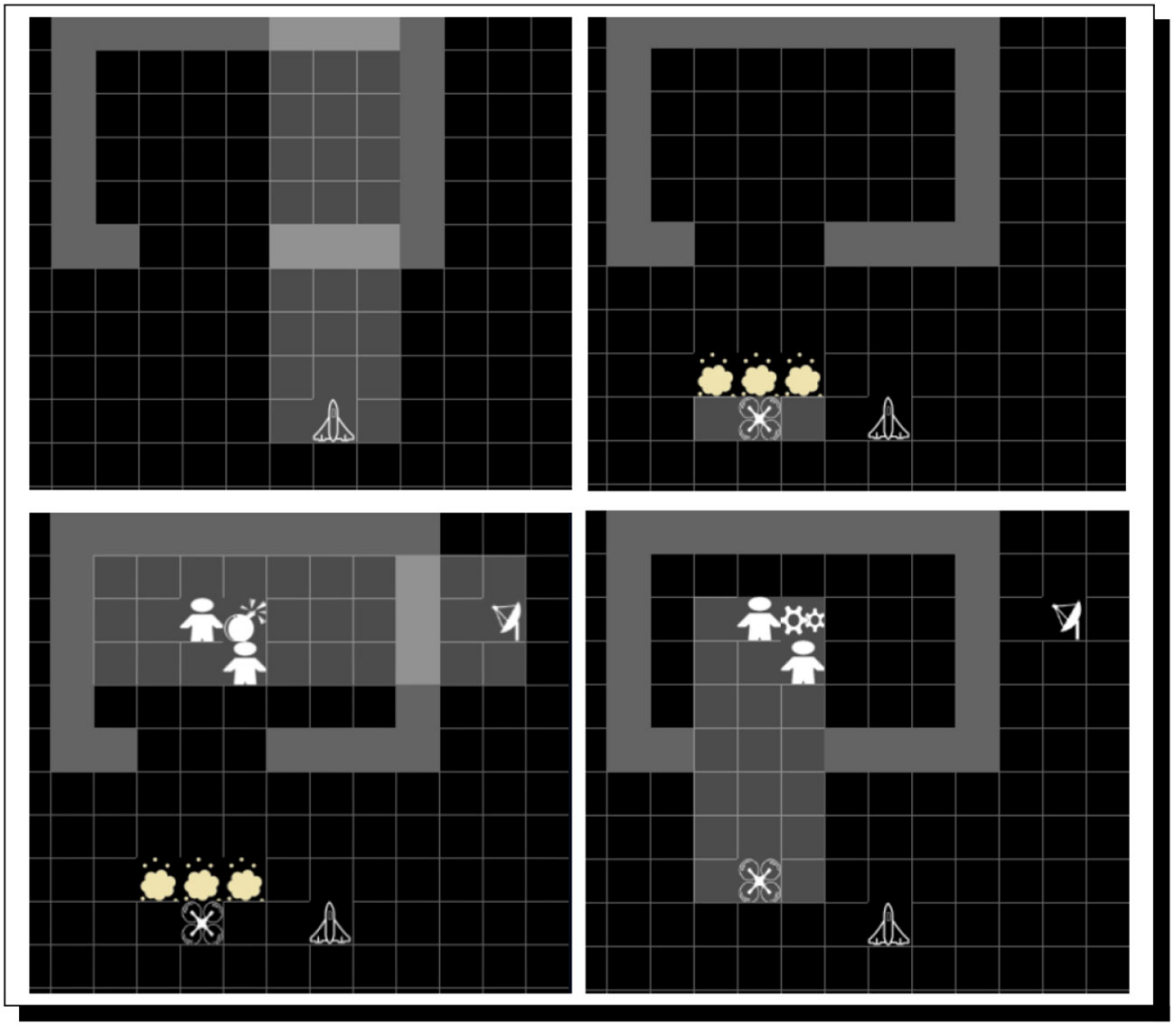
Multi-agent scanning. **(Top Left)** The high-altitude drone scans the building but does not perceive anything. **(Top Right)** The low-altitude drone moves in, but before attention can be focused on the objects in the building, a dust storm blocks its visual sensors. **(Bottom Left)** A ground-based agent with ground-penetrating radar moves into position and scans the inside of the building. **(Bottom Right)** The dust cloud disappears and the low-altitude drone's attention is drawn to the open building, where it perceives two men benignly working on an engine.

For implementation of this scenario, we use the Minigrid environment (Chevalier-Boisvert et al., 2018): a virtual grid world in which we can model our artificial agents with limited field-of-view and perceptual impedances. Our house is represented as a structure enclosed by walls that block visual sensors but allow use of wall-penetrating radar. There is an opening in the house; it represents a garage in which the individuals are working on the mysterious device. The individuals under investigation and the device being worked on are represented by special tiles, as are perceptual disturbances such as dust clouds. At a high level, the situation can be observed playing out in our environment, as shown in Figure 8. Our agents on the scene (i.e., *hdrone*, *ldrone*, and *radar*) use instances of ARCADIA, while the adjudicator agent (*adjudicator*), again, is built atop ShadowAdjudicator (Giancola et al., 2020), which now, courtesy of a tie-in with ARCADIA, has scientifically serious capacity for both perception and argument-based reasoning.^40^

**Figure 9:**
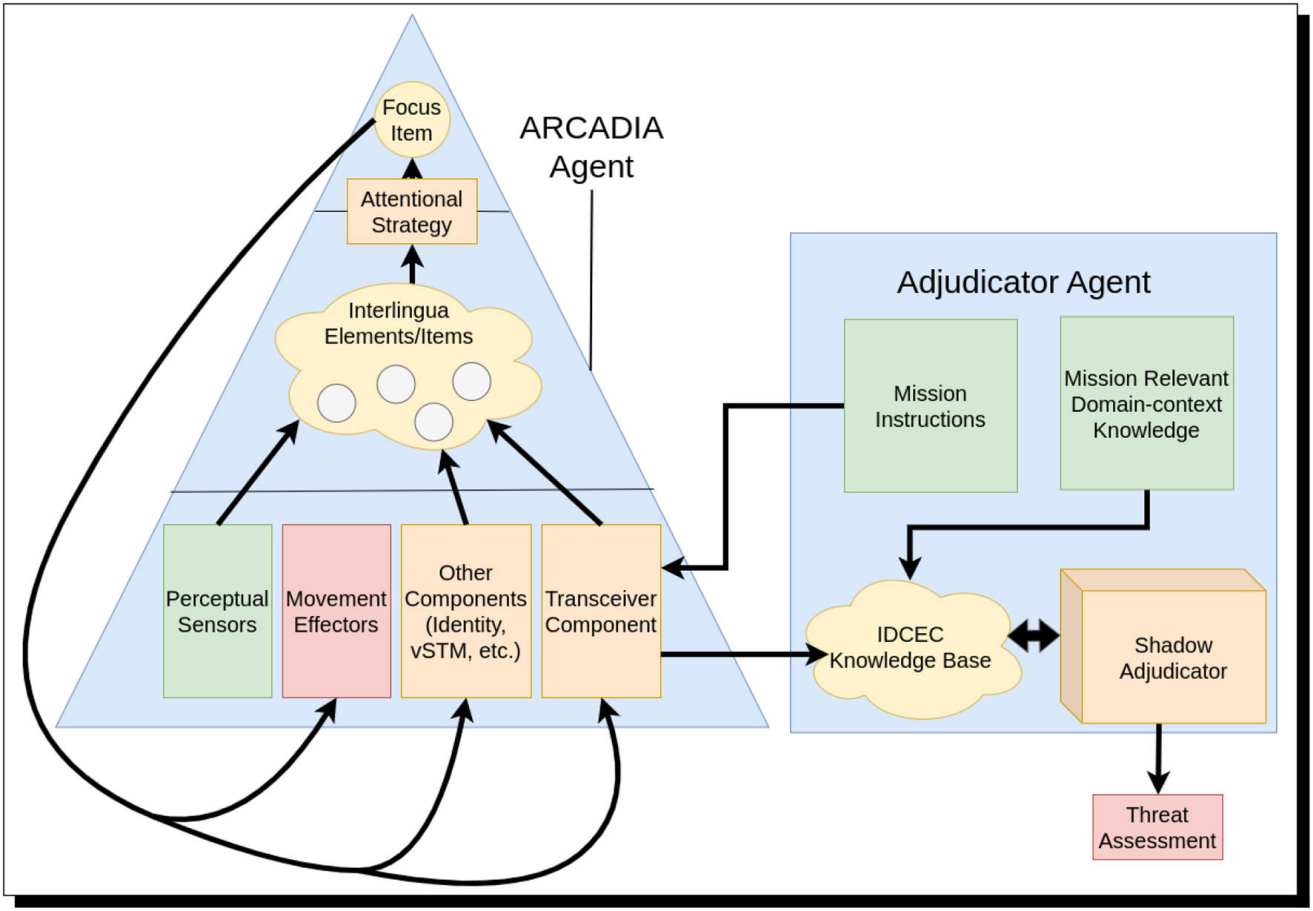
The information pipeline between the ARCADIA agent and the adjudicator agent. The high-level information pipeline between ARCADIA agents and the adjudicator agent is shown here. At each time step, mission instructions are passed to the ARCADIA agent in the situation via the agent's transceiver component. These commands are attended to and passed to the agent's movement effectors. The ARCADIA agent's perceptual sensors (visual, radar, etc.) pick out new items attended via the visual components that create objects. The finalized objects are interpreted to be fully perceived and are sent to the Adjudicator via the transceiver. The Adjudicator adjudicates between arguments factoring in the percepts of multiple agents on the ground, along with mission-relevant domain-context knowledge, to determine if there is a threat.

Time in our implementation is conceptualized as adjudication timesteps and ARCADIA steps. On each reasoner cycle, a predetermined movement command is issued to each of *hdrone*, *ldrone*, and *radar* by *adjudicator* and received by a transceiver component that creates an interlingua item based on this command. The attentional strategy prioritizes these command items; they, thus, become the focus of attention. The agent's movement effector component receives this command item and executes it. In parallel to this, ARCADIA's robust attentional-and-visual system monitors for changes from the visual sensor; this sensor creates items from objects in the field of view. In the event a fully represented object in memory is perceived and becomes the focus of attention, it will be passed to the transceiver component, which will, in turn, send a message containing the agent's perception to the *adjudicator* agent, which adds the information about the agent's perception to its knowledge-base. This information includes whether a threat was perceived or not. After receiving a new percept, *adjudicator* will reason over the known percepts and return a belief about the situation, in particular, the degree of belief regarding whether a threat exists. The overarching pipeline is shown in Figure 9. These degrees correspond to the levels introduced earlier in the present study (see again, if needed, Table 1).

**Figure 10:**
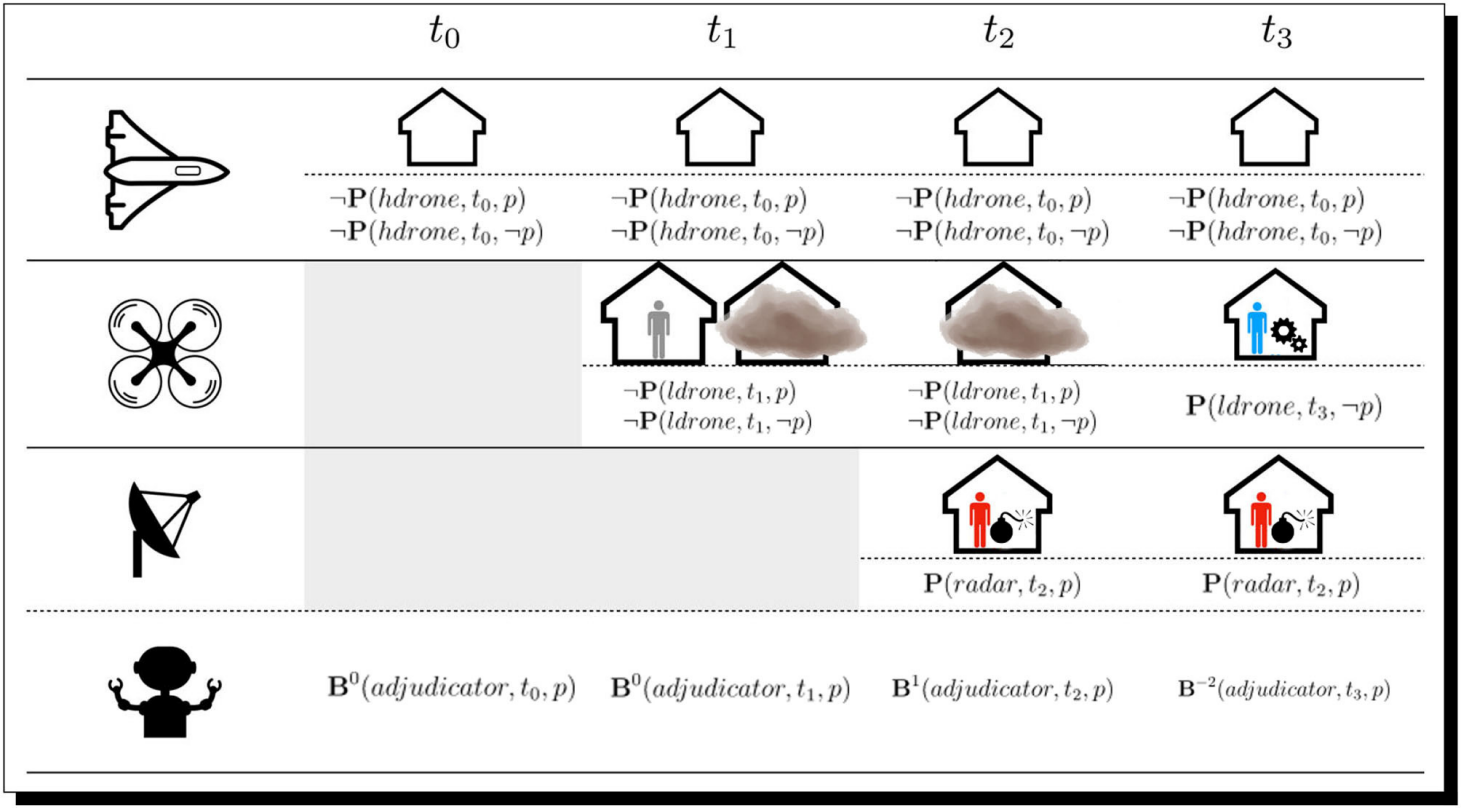
The timeline. Shown here is the state of the situation at each reasoner time-step, with the percepts of three agents in the scene regarding the proposition *p*, that a threat is present, where *p* is affirmed by the *adjudicator* if a dangerous object is reported as perceived. The adjudicator agent, on the bottom, reports its derived belief from the percepts at each reasoning time-step.

The situation plays out as follows and is presented in Figure 10. First, *hdrone* is issued orders to scan the building in a fly-by. It perceives the building but does not perceive any objects beyond this. From these percepts (or lack thereof in this case), *adjudicator* cannot determine whether there is a threat at this time-step, derived as a *counterbalanced* (recall again Section 7.1 and Table 1) belief as to whether or not there is a threat. In other words, at this point *adjudicator* is agnostic.

Next, the low-altitude drone (*ldrone*), in possession of a camera, receives orders to make an approach. As it obeys, its attention is focused on the people of interest and the device, but before the internal representation of the object can be fully assembled … a dust storm is kicked up, and this blocks *ldrone*'s visual sensors, which, in turn, nullifies its ability to have its visual component form representations of individuals or the device. Instead, it directs its attentional focus at the dust cloud itself; this blocks its view. These percepts of the people and cloud are sent back to *adjudicator*, which, at this point (rationally), maintains a *counterbalanced*/agnostic epistemic attitude regarding a threat/no-threat (i.e., re. *p*).^41^

At this point, the aforementioned ground-based agent with wall-penetrating radar (*radar*) is deployed to the side of the building. Its attention is drawn to two men located around the suspicious device. The ground-based agent reports these percepts to the adjudicator agent; it, accordingly, believes that there is *more likely than not* a threat present.

We explain in some detail the reasoning at *t*_2_ below. The adjudicator uses its Domain_Knowledge, which contains general rules for the situation, such as how to prioritize the beliefs of each agent and the definitions of negative and 0 belief in this context. When combined with the percepts reported by the ARCADIA Agents (IDCEC_KB_at_t2), ShadowAdjudicator is able to use IDCEC inference schemata to derive the current threat level. More formally, where this notation is simply “pretty printed” from underlying code, the situation is as follows:


$$\begin{array}{l} Domain\_Knowledge=\{\forall t_{0},t_{1},t_{2}:B^{h}(hdrone,t_{0},\phi )\\ \;\wedge B^{r}(radar,t_{1},\phi )\wedge B^{l}(ldrone,t_{2},\phi )\Rightarrow \\ \;B^{max(r\cdot 1/4,h\cdot 1/4,l\cdot 1/2)}(adjudicator,max(t_{0},t_{1},t_{2}),\phi ),\\ \;\forall t:B^{\sigma }(adjudicator,t,\neg \phi )\}\\ \;\Leftrightarrow B^{-\sigma }(adjudicator,t,\phi ),\\ \;\forall t:\forall a:\neg P(a,t,\neg \phi )\wedge \neg P(a,t,\phi )\Rightarrow B^{0}(a,t,\phi )\}\\ IDCEC\_KB\_at\_t2=\{\neg P(hdrone,t_{0},\neg p),\neg P(hdrone,t_{0},p),\\ \;\neg P(ldrone,t_{1},\neg p),\neg P(ldrone,t_{1},p)\\ \;P(radar,t_{2},\neg p)\}\\ Domain\_Knowledge\cup IDCEC\_KB\_at\_t2{⊢}_{IDCEC}\\ \;B^{1}(adjudicator,t_{2},p) \end{array}$$

Finally, the low-altitude drone (*ldrone*) manages to emerge from the dust storm after new orders and is thus once again able to observe into the building. It focuses its attention on the device and … perceives it to be a benign car engine. Once this information is relayed back to *adjudicator*, it reasons that it is *unlikely* there is a threat.

It should be noted here that *adjudicator* has situation-dependent definitions within its knowledge-base and is able to perform perception-infused reasoning that factors in these formulae. For example, notably, the true percept reported to the adjudicator is not really the presence of threat proposition *p* as simplifyingly shown in **P**(·, ·, *p*), as shown in Figure 10, but rather a percept of the true object that the agent perceives [in this case that of *hdrone*, **P**(*hdrone, t*0, *wall*)]. From this, *adjudicator* uses domain-context knowledge with the given percept to determine whether the agent perceived a threat or if not enough was perceived to ascertain whether the agent perceived a threat or not. Additionally, this extends to the *adjudicator* having a context-aware understanding of different types of agents and different levels of perception power, some being stronger than others, which is why the visual sensor on *ldrone* overrides the perceptions from *radar* at *t*_2_ and *t*_3_. This event leads to its final belief at the *unlikely* level. This is also why the percepts from the wall-penetrating radar only lead to a *more likely than not* level of belief, rather than a belief at the level of *likely* at *t*_2_.

Summing up, our third case study provides not only a potential real-life example in which our automated argumentation systems play a central and salutary role, but also demonstrates that our system has many capabilities outlined in our desiderata, **Des**. In particular, Case Study 3 exemplifies the defeasible nature of our system as encapsulated by desiderata *d*_1_ and the ability of our system to reason over cognitive operators as stated in desiderata *d*_6_. Regarding desiderata *d*_1_: As new information comes to light over the course of the scenario, the adjudicator is able to update its reasoning regarding the threat level at each time-step (see Figure 10); hence the reasoning capability of the system is observably defeasible, as desired. For desiderata *d*_6_, the system reasons over the cognitive operators for both belief and perception, as observed in depictions of both the agents on the scene and the adjudicator agent; see both Figure 10 and our presentation of Domain_Knowledge. This reasoning over cognitive operators also includes reasoning over the belief levels; hence part of desiderata *d*_3_ is satisfied.

## 8 Sophistic argumentation

There is, it seems to us, a long-standing bias or presumption within the logicist AI tradition (into which, as explained above, our study as reported herein firmly falls) that treats arguments as fundamentally similar to earnestly constructed proofs (or at least to simplified, scaled-down proofs, earnestly and sincerely constructed). In this tradition, the purpose or function of arguments, like that of the authoring of proofs by humans engaged in the formal sciences, is to support rational belief fixation and to thereby enable new knowledge to arrive in the mind of cognizers who assimilate these proofs. This tradition makes room for and indeed realistically expects (at least periodically) invalid proofs (the history of mathematics having seen many), just as the tradition of computer programming makes plenty of room for programs that are invalid (but certainly programs).^42^ In point of fact, we ourselves, in adopting a thoroughgoing inference-theoretic perspective, regard arguments to be akin to proofs and argument crafting on the part of humans to be akin to the craft of articulating proofs. However, while arguments do often function as demonstration and warrant in support of belief and decision-making, these are undeniably neither the sole functions of arguments nor are all warrants rational ones. This is something we suspect that AI should start to take note of, carefully. We, thus, now briefly explain, and our explanation will wrap up by drawing once again upon the three-door Monty Hall Problem = MHP_3_, now familiar to our readers given earlier discussion of this problem.

To explain, let us first consider the function of arguments: Arguments are often instruments of persuasion. In fact, an argument's persuasiveness may be of greater import than its veracity or validity, depending on the arguer's intent with regard to its audience. Logicist AI has largely followed in the footsteps of formal logic by privileging the dialectic (i.e., in a word, logic) over the other members of the ancient trivium. By eschewing rhetoric (essentially argumentation as treated today what is known as *informal logic*; see Groarke, 1996/2017), most logicist AI fails to appreciate the persuasive function of argument and its role in dialogical games such as disputation. This failure is not a small one. The persuasive power of argument is central to the practice of policy-making, politics, and law, and the life-altering decisions sometimes made therein. Moreover, persuasion is essential to the utility and success of logicist AI—even if this is unrecognized by practitioners. Why is it essential? Well, insofar as logicist AI in support of, and interacting with, humans is concerned, the goal is both to “be correct” and to “be believed;” systems that are correct but not believed are useless. Furthermore, we charitably assume that acceptance and use of these logicist-AI systems are intended to be volitional, and as such, the goal again is to “be believed,” not simply to “be obeyed;” systems that are obeyed even when not believed are undesirable, dangerous, and potentially unethical.

Second, regarding rationality, arguments can be persuasive even when they are invalid or untruthful, and veracious arguments can be unpersuasive (as the literature on MHP_3_ confirms; see the discussion of this empirical fact in Chapter 1 of Pinker, 2021). In terms of bringing about human belief, validity and veracity guarantee nothing. That invalid, pseudo-rational arguments can be persuasive is not a new revelation; Aristotle knew this over two millennia back when he wrote that arguments can have the appearance (but not always the substance) of demonstrable justification that makes belief warranted (Aristotle, 1823). Indeed, the methodological and disciplinary distinction between rhetoric and dialectic—between persuasion and veracity—dates back firmly and in general to ancient Greece and the age and work of not just Aristotle, but Plato and Socrates (see, specifically, the claimed intellectual battles between Socrates and the sophists).

Who were the sophists? To brutally summarize some of Plato's dialogues, the sophists were itinerant teachers who, for money, taught the skill of persuasive argumentation and debate to Athenian citizens so that they might prevail in the courts and in civic life—even if they were in the wrong. The sophists were criticized and opposed by Socrates and others because they (supposedly) only cared about being persuasive. They have been characterized as purveyors of the semblance of wisdom and not the genuine article, having rejected the doctrinal ideal of “truth” to promulgate, instead of the virtue of persuasive cleverness without moral good (Aristotle, 1955). While many contemporary scholars (see Marback, 1999; Gagarin, 2001; McComiskey, 2002) have attempted to rehabilitate the sophists' reputation, the legacy of the sophists—among both scholars the general public—still amounts to “sophistry” being a byword for insincerity, self-interest, and, above all, manipulative persuasion by clever argumentation.

This encapsulated history of the sophists is given by us here for more than just trivia; the sophists demonstrated the power and importance of persuasion (viz. rhetoric), attempted to systematize it, and stand as a cautionary warning about the pursuit of argument-based persuasion unchecked by truth or virtue. However, why, the reader might ask, did the sophists' techniques work? More importantly, why are invalid arguments sometimes so persuasive? The answer to that is rather simple: Absent sufficient training and in-the-moment mental effort, humans are abysmal at normative argumentation and rational judgment. Humans are, unknowingly, imperfect reasoners who predictably and instinctively succumb to a host of biases and illusions and, moreover, are supremely, yet undeservedly, overconfident of their ability to reason and judge the reasoning of others—at least when compared with the standards of formal deductive and inductive logics and probability theory.

Moreover, the takeaway is that not only do logicist-AI systems need argumentation but also they need persuasive argumentation that ensures and preserves truthfulness (veracity) and formal validity in order to engender rational human use. Perhaps the reader will agree that we do not want artificial agents able to understand and generate arguments wonderfully, in order to, in part, persuade humans sophistically.

Before moving on to the final section of the present study, it is, in our view, worthwhile to say a bit more about the sophists, and to then end this section by looking at a specimen of just the sort of sophistic argumentation that AI systems should not produce and promote in order to persuade humans.

Naïve and unfair as their remembrance may be—the truth is that ancient sophistic techniques have been vibrantly alive and well and continuously refined for over two millennia—persuasive techniques that prey upon the audience's cognitive dissonance, ignorance, intellectual laziness, and desire for comforting belief reinforcement. Is there the specter of digital sophists emerging? Why yes. Sophistic AI is literally a past accomplishment. Starting in the early 2000s, the application of AI to natural argumentation refocused on audience-centric systems that take subjective aspects of argumentation seriously (see Reed and Grasso, 2001, 2007; Reed and Norman, 2004) and this resulted in the development of various neo-rhetorical (e.g., Grasso, 2002) and logico-dialectical (e.g., Aubry and Risch, 2006) approaches to persuasive and deceptive argumentation. In 2010, cognitive models were added to the mix, resulting in *The Lying Machine* (Clark, 2010), an explicitly sophistic artificial agent that persuades via a combination of argumentation and illusion.

The Lying Machine (TLM) is a logicist-AI system that manipulates human beliefs through persuasive argument by using cognitive models to generate convincing yet potentially disingenuous arguments. In design, the machine maintains conceptually separate repositories for its first- and second-order beliefs (i.e., its beliefs about the world and its beliefs about its audience's beliefs about the world). It reasons over first-order beliefs in a normatively correct fashion, but when reasoning over second-order beliefs, it uses both normatively correct reasoning and a predictive theory of human reasoning, namely, *mental models* theory (Johnson-Laird, 1983, 2006), one of the most influential theories of human reasoning in cognitive science. In so doing, the machine internally contrasts (i) what it believes, (ii) what it believes its audience ought to believe were they to reason correctly, and (iii) what it believes its audience will likely believe given their predicted fallibility. In operation, TLM seeks to achieve various persuasion goals of the form “persuade the audience of ϕ,” where ϕ is a logicization of a proposition 〈ϕ〉 about the world. Given such a goal, the machine first forms its own justified belief about ϕ.^43^ TLM, then, determines whether its audience ought to believe 〈ϕ〉 and whether 〈ϕ〉 can be justified in convincing fashion based solely on second-order beliefs (i.e., beliefs it ascribes to its audience). If so, the machine, then, constructs and articulates a credible argument for ϕ, presented then as an argument for 〈ϕ〉.^44^ Like the sophists, TLM aims for *perceived* credibility as opposed to objective, logical, or epistemological credibility. While its arguments may be logically valid or invalid, the importance is that they *appear* valid to its audience. Argument credibility is enhanced by limiting the initial premises to what the audience is believed by TLM to already believe. Moreover, since the machine is not constrained by logical validity, it is able to produce all of the following types of arguments:

- a veracious argument for a true proposition emanating from shared beliefs;
- a valid argument for a false proposition emanating from one or more false premises that the audience erroneously believes already;
- a fallacious argument for a true proposition (an expedient fiction for the fraudulent conveyance of a truth); and
- a fallacious argument for a false proposition (the most opprobrious form being one that insidiously passes from true premises to a false conclusion).

With the above repertoire in hand, the lying machine attempts to take on the pejorative mantle of the sophists by causing arbitrary belief to materialize in the minds of those targeted, through persuasive argumentation without concern for validity, sincerity, or truth. The results of experiments with TLM are, perhaps, unfortunate but not surprising, given that the fully replicated and thoroughly confirmed empirical fact of the matter in the cognitive science of reasoning has disclosed that humans confidently believe any number of things on the strength of reason that is often downright absurd, logically and mathematically speaking. [An excellent, if depressing, survey of this science is given in the study by Pinker (2021), the anchoring first chapter of which features the very same MHP_3_ problem first introduced in the present essay in Section 2.] Humans find the machine's sophistic arguments both credible and persuasive, even when those arguments are opposed by (logically) valid rebuttals (Clark, 2010, 2011).

We now end the present with an informal presentation of an argument regarding MHP_3_ that practitioners of human-centric AI need to ensure is not generated, nor accepted, by artificial agents. The argument in question is in support of a policy of stay in the problem, and runs as follows:

**The Lame-Horse Argument**

(1) Suppose you bet at random on Horse #2 in a three-horse race, where all three horses at the outset are indistinguishable with respect to all of their respective racing-relevant properties.
  (Of course, the idea is that in MHP_3_ we have a three-door “race,” and the bet is the initial selection of one of the three doors.)
(2) From (1), we deduce that your odds of winning at *t*, the moment the race starts, are $\frac{1}{3}$.
(3) Suppose as well that during the race, at *t*′(*t*′ > *t*), Horse #3 suddenly comes up lame and is out for good, while Horse #1 and Horse #2 continue running, neck and neck.
(4) From (3), we deduce that your odds of winning at *t*″(*t*″ > *t*′), the moment after Horse #3 drops out, are ½.
(5) We can also infer that switching your bet to Horse #1 at the next instant *t*^‴^(*t*^‴^ > *t*″), with all conditions remaining the same (& assuming that you are given the opportunity to switch) is irrational, because the effort of doing so will not improve your ½ odds at all.
(6) Since the scenario here is isomorphic to that seen in MHP_3_ (where of course your opportunity to switch doors is just like your opportunity to switch horses), it's irrational for you, or for that matter any contestant, to switch doors after Monty Hall reveals a donkey (or llama, etc.), a move that is of course the analog for Horse #3 coming up lame and thus “revealing” itself to be a guaranteed loser.

The Lame Horse Argument is a powerful sophistic argument; as Pinker (2021) explains, it even persuaded many professional mathematicians that a stay policy in MHP_3_ is irrational (an extensive treatment of, and references for, The Lame-Horse Argument, can be found in the study by Granberg, 2014). Of course, this is not to say that such mathematicians *intended* to persuade their targets while knowing that their argument was invalid. However, regardless, this is certainly something that could be done by malevolent agents (whether human or artificial), rather easily. Thus, if we may be so bold, the argument here is one that by our lights, the sophists would be quite happy with, in general; it is an argument, if you will, right up their alley.

However, *why* is The Lame-Horse Argument unsound? Though it is persuasive, it is not veracious because (in short), in point of fact, the two scenarios are not isomorphic at all (and that they are is a premise in the argument); they are not even analogous by the simplest inference schemata for analogical argumentation.^45^ The reason is that a number of intensional factors in the mind of Monty Hall himself are crucial to a correct, reasoned solutions, but these factors are entirely absent from the three-horse scenario; these factors were discussed and logicized in the cognitive calculus DCEC in Section 7.1.^46^

## 9 Next steps; conclusion

We now briefly describe a series of steps we are already in the process of taking, to further broaden and apply our approach. Readers both alert and knowledgeable will in the case of most if not all of them have already wondered whether our approach is applicable in these directions.

### 9.1 Surmounting the paradoxes of perception

The history of argument-based defeasible/non-monotonic systems in AI, as evidenced prominently by Pollock (1995), has been driven in no small part by the need to solve certain paradoxes, among which are the Lottery Paradox and the Paradox of the Preface.^47^ Are there paradoxes specifically in the intersection of perception and such argumentation systems? Indeed there are; see for example the rather tricky one presented in Davis (1989). We are working hard on proving, and empirically demonstrating via simulations, that this and other even-harder paradoxes can be surmounted by our cognitive calculi and associated automated reasoners, in keeping with the desiderata that sum up our approach.

### 9.2 What about abductive argumentation?

Some of our readers will inevitably be curious about a type of reasoning we have yet to touch upon: *abductive* reasoning.^48^ While it is certainly the case that there is no consensus as to what the precise nature of this reasoning is, the agreed-upon kernel of such reasoning in formal logic and AI expressed as an inference schema at least roughly in the fashion, followed earlier in the study, is as follows (where “ϕ” and “ψ” are formulae in accordance with some formal language, ‘ν” denotes one or more variables free in these formulae, and χ denotes one or more constants/names):


$$\begin{array}{l} \frac{\psi \left(\chi \right),\forall \nu \left[\phi \left(\nu \right)\to \psi \left(\nu \right)\right]}{\phi \left(\chi \right)} \end{array}$$

Let us label this inference schema “*I*_*A*_.” This (deductively invalid, as desired) schema accords with many of the simple, familiar specimens of abduction. For example, suppose that soon after waking in the morning Bertram goes to the kitchen to make a cup of coffee, but upon entering the room finds a steaming cup of cappuccino sitting on his placemat at the breakfast table. No one else is present. Bertram asks himself: How did this situation come to be? Knowing that there is only one person—Abigail—in his household fully capable of making the exact kind of coffee he prefers, with knowledge of where he customarily sits, Bertram abduces via *I*_*A*_, instantiated, to produce the following argument, to which Bertram accedes, and the mystery is solved (and he has gained knowledge as to whence the coffee cup).^49^

**Table:** The Abductive Coffee-Mystery Argument

∴	1.	*OnTable*(**cup*22*)
	2.	*Prepared*(**abigail, cup*22*) → *OnTable*(**cup*22*)
	3.	*Prepared*(**abigail*, *cup*22*)

Unfortunately, as has been long and widely appreciated, *I*_*A*_, and indeed any schema that is of this general sort, is deeply problematic. The set of defects has little to do with the mere (and desired) fact that abductive reasoning is non-deductive (it is, in this regard, a specific type of reasoning falling with inductive logic as the subdiscipline of logic our work falls into and is hence analyzed in the study by Johnson, 2016). For instance, this set of defects includes the havoc that can ensue from multiple uses of *I*_*A*_: Let the universally quantified formula be instantiated twice (separately) to yield


∀x[R(x)→S(x)]

and


∀x[¬R(x)→T(x)],

and then suppose we have *S*(*a*) and *T*(*a*). A contradiction is, then, directly provable by two inferences, each in conformity with *I*_*A*_.

Thus, one can view the chief challenge of working out a logic of abduction in the style of our cognitive calculi to be specifically the development of inference schemata that (i) are in the spirit of *I*_*A*_, (ii) are (as it in fact is) machine checkable so that abductive argumentation is verifiable/falsifiable but (iii) have none of the obviously objectionable attributes of this inference schema. Of all the work we are aware of in this vein, Meheus and Batens (2006) comes closest to conforming to it and our approach. In this study, there is firm insistence upon having a proof theory, indeed one that is based on an attempt to expand and refine *I*_*A*_. However, this proof theory could not be used to model and solve any of our three case studies. The reason is that the logic in question, **LA**^*r*^, is purely extensional, as admitted by the researchers in question:

The logic presented in this study [**LA**^*r*^] will be based on Classical Logic — henceforth **CL**. Moreover, all references to causality, laws of nature, and similar non-extensional concepts [such as belief, knowledge, and perception] will be out of the picture. We do not doubt that more interesting results may be obtained from intensional logics (Meheus and Batens, 2006, p. 22–223).

This quote can be viewed as a convenient stepping stone for a next step on our part, in which our cognitive calculi and automated reasoners, as introduced, explained, and deployed above, cover human-level abductive argumentation. The novel inference schemata in these calculi will minimally have perception and epistemic operators. Additionally, there would be a knowledge-base for the agent/s reasoning abductively. Thus, from our perspective, the coffee mystery is an enthymematic argument, both perceptually and epistemically. To achieve more precision, schema *I*_*A*_ would need to be expanded and refined; here, in fact, is a schema—$I_{A}^{\text{int}}$—marking a first such step in that direction, making use of the operators **B**, **K**, and **P** (for, as the reader will recall from the foregoing, belief, knowledge, and perception, respectively):


$$\begin{array}{l} \frac{\text{P}\left(𝔞,\psi \left(\chi \right)\right),\text{K}\left(𝔞,\forall \nu \left[\phi \left(\nu \right)\to \psi \left(\nu \right)\right]\right)}{\text{B}\left(𝔞,\phi \left(\chi \right)\right)} \end{array}$$

This inference schema can formally and computationally undergird the argument Bertram might offer to someone as to why he regards the “mystery” to be solved, the idea being that he would express his reliance on *perceiving* the cup of cappuccino and his *knowing* beforehand the key conditional formula (and particular propositions re. Abigail), suitably instantiated. We are actively working on the expansion of our paradigm in this abductive direction.

### 9.3 What about pictorial argumentation?

Human agents make considerable use, even in sophisticated settings observed in the formal sciences, of arguments and proofs that include *pictorial* representations, where such representations are not reduced, and in some case not even in principle reducible to, symbolic content. [In our study described above (Case Study 2), we have of course relied on the reduction of diagrams in Meta-Forms to linguistic formulae.] Notably, we are not here referring to arguments or proofs laid out in graphical ways (an important issue briefly discussed in Footnote 17). Reasoning frameworks, at least of the deductive sort that subsume extensional logics such as L_1_ and include both symbolic content (e.g., formulae in the formal language of a logic or—as in our case—cognitive calculi) and pictorial content, were seminally introduced by Barwise and Etchemendy (1995); they call such logics *heterogeneous*. Subsequently, a more general formal logic for heterogeneous reasoning, Vivid, was introduced by Arkoudas and Bringsjord (2009b). Vivid can be used to allow PERI.2 (and for that matter any logicist artificial agent) to reason about the Meta-Forms game board and clues relating to it as a diagram, unreduced to or represented by anything linguistic/symbolic. We are actively working on this direction, based on a new cognitive calculus with all the extant expressive and reasoning powers of DCEC and IDCEC and, at the same time, the vivid-like capacity to directly and irreducibly represent and allow reasoning over pictures, images, and diagrams.

### 9.4 Final words

We end by admitting that, at least in our view, the most daunting obstacle standing in the way of HCAI being based on argumentation science and engineering is not a technical one, at all. We are, for what it is worth, completely confident that the research trajectory explained (and hopefully rendered at least somewhat promising in the reader's view by virtue of the foregoing) above can indeed be used as the basis of artificial agents with near-human-level intelligence that profoundly help humans. However, humans have to *want* what argumentation-centric AI can provide. Our directive **Dir** is not (yet) universally affirmed. In a world where forms of AI, for instance large language models produced by so-called “Deep Learning,” wholly forego any argument or proof of the sort that we are calling for, we see room for plenty of rational concern. The forms we refer, as the reader will likely well-know, are purely statistical/connectionist ones entirely devoid of any declarative content expressed in accordance with a formal language (since they rely upon tokenization into formats that are only strings with none of the structure of quantification, inference schemata, etc.) and thus by definition devoid of any reasoning over such content in accordance with inference schemata.

## Data availability statement

The raw data supporting the conclusions of this article will be made available by the authors, without undue reservation.

## Author contributions

MC an expert on AI and sophistic argumentation and the automation thereof, principally contributed the vast majority of the section on this subject, and ensured that prose relating to this topic was suitably integrated across the essay. MC made contributions to many additional facets of the paper as well. PB chief architect and developer of ARCADIA, oversaw and coordinated all integration between attention and perception on the one hand (as modeled in ARCADIA), and automated reasoning and automated planning on the other. He made contributions to many other facets of the paper as well. JO is one of the designers and principal writer of the ARCADIA-based case study, and leveraged his understanding of both ARCADIA and the RAIR Lab's automated reasoning/planning assets as well to engineer and describe the MiniGrid case study. JO also made contributions to many other facets of the paper. JS is the cognitive roboticist on the team; PERI.2 is overseen, engineered, and managed by him and his team in the RAIR Lab. Accordingly, the robot case study was enabled by JS and his efforts. JS made contributions to many other facets of the paper as well. NG is the long-time principal architect and developer of automated-reasoning and automated-planning systems in and from the RAIR Lab; in the case at hand, he originated the designs and code for both ShadowProver and Spectra (the former being part of the foundation of ShadowAdjudicator). NG is also the inventor of a number of cognitive calculi referred to in the paper. Overall, NG's work enables and infuses nearly all facets of the paper. MG with SB, was the principal writer of the paper, is the lead developer of ShadowAdjudicator, wrote with SB the “manifesto” part of the paper and propagated it throughout the essay, and used his expertise on MHPk to provide crucial content throughout the paper. MG worked directly on nearly every part of the paper, start to finish, engineered runs of ShadowAdjudicator and ShadowReasoner, and archived and presented parts of these runs in the paper. SB is the inventor of the first cognitive calculi, and, at least in part, of every cognitive calculus since the first appeared early in the 21st century. He worked with NG on automated reasoning and planning to lay the foundation for the project here, before it started. SB is an expert in computational inductive logic and defeasible inductive reasoning, cognitive likelihood (which he originated), conceived and designed the paper, wrote early drafts of it, and continued to write/edit nearly all content in subsequent drafts, through to the final version.

## Footnotes

^1^As well as proofs, which we take to be just a special case of arguments. Abstractly put, an argument for us is a sequence of formulae in some formal language of some logic or logics, where the sequence links these formulae by instances of inference schemata. A proof is an argument in which (i) the inference schemata in play are restricted to deductive ones, and (ii) some premises given in the sequence in question enjoy special status because they are members of a pre-identified axiom system (e.g., axioms for Euclidean geometry, or for arithmetic, or topology).

^2^MHP, as a matter of fact, in our formalization and solution, involves *both* likelihood and probability. Since the emphasis, herein, is very much on the former, we do not bring to bear our full formalization and implementation of the probability calculus of Kolmogorov (1933) within a richer version of IDCEC. Doing so would be overkill in the present study, since the key manner of handling uncertainty is here cognitive likelihood not probability.

^3^A modern translation into English of Aristotle's writings.

^4^A nice, modern overview of this fragment of first-order logic = L is given in the study mentioned in Smith (2017).

^5^The Spielberg–Kubrick film *A.I* includes a rather depressing depiction of a number of humans who are, in fact, like Jones. We refer to the (disturbing) stretch of the film in which humans destroy robot after robot in violent, sadistic fashion.

^6^This not being a study on formal verification, we omit formal verification.

^7^Exactly parallel points as we make in the present section could be made about GPT-4, discussed (and greatly lauded) in the study mentioned in Bubeck et al. (2023). The details behind GPT-4 are unfortunately proprietary; Meta has made available to all its transformer infrastructure.

^8^See Tierney (1991), and for a shorter historical account, Chap. 1 of Pinker (2021). For fuller discussion of the mathematicians to whom we have just referred, see Granberg (2014).

^9^A view affirmed and used in the Prolog-centric (and hence only extensional) and pedagogically oriented tour through thinking as reasoning in the study mentioned in Levesque (2012).

^10^The lead author, along with author P. Bello, is, in addition, convinced not only that an agent's perception of its internal, mental environment is equally important, but also working on formalizing and implementing the relationship between internal perception (which calls for its own intensional operator in cognitive calculi) and reasoning. For an exploration of internal perception in self-conscious robots that is integrated with robust reasoning in a cognitive calculus, see Bringsjord et al. (2015).

^11^Ancestors and less-developed lists of the desiderata in question have been given in some previous studies, including e.g., Bringsjord et al. (2020a).

^12^A comprehensive history, in our opinion, needs to be composed by someone at some point. Part of the challenge is the need for the brave author who takes this project to be fluent in at least both ancient Greek and German. The former language is key because Aristotle can be viewed as the primogenitor (e.g., see *Topica* and *De Sophisticis Elenchis* in his *Organon*, available in McKeon, 1941). German is crucial because, even to this day, the remarkable work of Lorenzen, momentarily discussed, has not been fully translated from the German. In addition, the lead author is personally of the view that the work of Leibniz in formal logic (which includes the long-before-Frege invention of both first-order logic and modal logic; see Lenzen, 2004), and in particular work toward his dream of a “universal logic” (which is expressly intended to cover the dynamic argumentation of multiple, interacting agents), is quite relevant to any full history of the sort we are imagining, which means that command of Latin and French also becomes a requirement for the relevant scholarship [we return to the Leibnizian nature of our paradigm below (Section 4.2)].

^13^We read as follows:
It has been seen that Dung's fundamental model, as described in [73], abstracts away such internal structure from individual argument in order to focus on the manner in which arguments interact *via* the defined attack relationship. In unfolding the exact nature of “the argument x attacks the argument y,” however, the *reason* why such an attack is present needs to be considered in terms of those *structural schema* underlying the arguments *x* and *y* from which the attack arises. Such an interpretation, therefore, raises issues that concern the form an argument might take, i.e. issues regarding the components and representation of *arguments* rather than the process and outcome of the *argumentation* involved (Bench-Capon and Dunne, 2007, p. 625).

^14^It is worth pointing out that Dung's (1995) abstract scheme for arguments is (unbeknown in our experience to most researchers working in AI and computational argumentation systems and formalisms) related to, indeed in some non-trivial respects anticipated by, a longstanding sub-area of formal logic that spans both extensional and intensional logic; we refer to *justification logic*. A nice starting point is Artemov (2008). The core idea in justification logic (to simplify rather harshly for economy) is that formulae that express some proposition, say ϕ, are accompanied by justifications, and we thus have for instance *t*:ϕ, where *t* is the justification. Justifications, here, have long been conceived as proofs and/or arguments. This tradition, and the logico-mathematical results that have been obtained, run back to a time (circa 1930 in the case of extensional logic, within mathematical logic) quite before the study by Dung and others on abstract schemes for the systematic study of argumentation. For a detailed overview, see Artemov and Fitting (2020) (while this overview credits some early mathematical logicians, e.g., Kolmogorov, 1932, with laying the foundations of justification logic because of their identifying “truth” with “provability,” it does not credit, as the first author of the present study feels it should, those who established proof-theoretic semantics, as also laying part of these foundations. As observed below when we present the technical basis of our approach to and work on computational inductive logic, proof-theoretic semantics has greatly influenced this approach/work). Regarding our own approach, the lack of internal structure in justifications in justification logic, which parallels the situation with respect to Dung's approach, means that our computational logics and AI systems for argumentation-centric AI are very different. This is expressed explicitly in desiderata *d*_4_ and *d*_5_ in our set **Des** of desiderata, given in Section 5.

^15^No quantifiers, but constants to denote individuals, unrestricted use of *n*-ary relation and function symbols, the identity = relation, and inference schemata for deduction using identity, e.g., that if *a*=*b* and ϕ(*a*) (a formula in which constant *a* occurs), then inference to ϕ(*b*) is permitted.

^16^For an efficient overview of defeasible reasoning, in general, the interested reader for whom defeasible/non-monotonic reasoning is new is directed first to the study mentioned in Koons (2017).

^17^There are at least two other important, substantive matters that must for economy be left aside, which are quite important. The first is that as a matter of fact, the arguments and proofs that are key to our study are often expressed in what is as far as we know a novel graphical form of so-called “natural reasoning”: *hypergraphical* natural reasoning because arguments, proofs, and semantic structures [e.g., a hypergraphical version of so-called “truth trees” (as nicely introduced in Bergmann et al., 2013)] are all expressed as hypergraphs (Berge, 1989; Bretto, 2013), including 3-dimensional hypergraphs; see Bringsjord et al. (2023a). We observed our hypergraphical approach as being within the general fold of graphical schemes for argumentation, a nice example of which is given in the study mentioned in Bench-Capon et al. (1992), which is, in turn, within the general approach of Toulmin (2003). A look at a robust hypergraphical proof within a logicized theory of special relativity that faster-than-light travel is impossible (see Govindarajalulu et al., 2015).

^18^Bench-Capon and Dunne (2007) astutely concede in footnote #2 on page 620 that even in mathematics there are disputes about premises (or axioms); they give the Axiom of Choice as an example. However, they insist that a *sine qua non* for rational dispute is having on hand an “alternative theory” (in this case e.g., **ZF** rather than **ZFC**). Moreover, at any given point in mathematics (and, needless to say, mathematical physics pursued through formal logic), there has been dispute in the absence of an alternative theory. A case in point is the rejection to Cantor's seminal introduction of transfinite numbers and their logic by many mathematicians, on the grounds not of an alternative theory, but of their perceived near absurdity. A more recent case in point is that Gödel's now-fulfilled prophecy that new axioms governing very large sets (e.g., the independence of the Continuum Hypothesis from **ZF**/**ZFC**) would simply be legislated. Another example, perhaps the sharpest one, is the rejection of infinitesimals in the absence of alternative theory that accommodated them (rather than simply leaving aside, as in the case of limits), and then the subsequent vindication of Leibniz on infinitesimals by Robinson (1996).

^19^Alert readers will perceive that our terminological practice in the present study reflects this, as e.g., we sometimes use “proof” instead of “argument” to refer to a chain of inferences found automatically by our ShadowProver system.

^20^For a discussion of this sort of explicit rigidity in the case of analogical inference, see Bringsjord and Licato (2015). Analogical inference schemata arise again below, in Section 8.

^21^The point in future time at which, so the story goes, AIs reach human-level intelligence, and then immediately thereafter ascend to intellectual heights far, far above our own.

^22^This is what allows proofs of the Halting Problem for Turing machines to be relied upon to prove the undecidability of the *Entscheidungsproblem*; see Boolos et al. (2003).

^23^As pointed out in that study, as far as most relevant thinkers know, it was actually Ray Reiter (the same thinker who introduced default logic, briefly mentioned above) who coined and first defined the phrase “cognitive robotics.”

^24^Bringsjord's rejection of possible-worlds semantics can be traced to his proof rather long ago that such structures can be shown to be mathematically impossible; see Bringsjord (1985).

^25^There will be the off case of a query, e.g., as to whether a low-expressivity ϕ is inferable from a low-expressivity Φ, for instance when all formulae selected for automated processing are mere propositional-calculus formulae, or—more realistically—when all formulae fall into a decidable fragment of L_1_, e.g., fluted logic. However, the standard cases for use of cognitive calculi, which are multi-modal quantified logics, will include high-expressivity formulae.

^26^In general, once one moves beyond first-order logic, a dramatic loss of metatheoretical properties desirable to many (not us) occurs, as revealed in Lindström's Theorems, elegantly covered in Ebbinghaus et al. (1994).

^27^When the extensional core is L_3_.

^28^We do not spend the space to recount why.

^29^In fact, every run arising from every query that triggers automated reasoning/planning in our three case studies is clocked in milliseconds: no run exceeded 3 s on an Apple laptop.

^30^We do not by the way mean to imply that no one within the ASP rubric has tackled human-level cognition. Ganascia (2007), e.g., has modeled and simulated aspects of lying constrained by this rubric.

^31^Gödel's results pertain directly only to elementary number theory, but they carry over their application to other domains.

^32^Following long-established and customary terminology, a *sound* argument is both *veracious* and *valid*; a veracious argument has true (or at least plausible to some level in some stratification of plausibility) premises; and a valid argument has inferences that abide by the collection of inference schemata taken to be operative in the case at hand.

^33^Since it is equally likely that the prize is behind any of the three doors, the same argument can be generated regardless of which door is initially selected.

^34^See Pinker (2021) for an argument (authored and advocated by a mathematician) made by a analogy to horse race, and for more extensive coverage of such arguments, see Granberg (2014). We explain in the study mentioned in Section 8 that two-horse arguments are ideal specimens of sophistic argumentation.

^35^To the best of our knowledge, while the first multi-valued modal logic (a three-valued one) appeared in 1967 due to the study by Segerberg (1967), IDCEC is the first multi-valued *multi*-modal-operator logic, and with little question (for better or worse), the first such logic computationally implemented. Multi-valued logics in the non-modal/extensional case (three values) originated with the study by Łukasiewicz (1920), and our basis on the extensional side (e.g., standard L_1_) for our cognitive calculi is an extension and refinement of Łukasiewicz's study by Kleene's (1938); see again desideratum *d*_3_ in **Des**.

^36^This approach to probability can be formalized in what is known as *probability logic* (Adams, 1998), and probability logic can be subsumed in cognitive calculi that subsume the two—DCEC and IDCEC–we employ herein, but this is out of scope.

^37^The precursor robot, PERI, anchored the introduction, to the field of AI, what is called *psychometric* AI; see Bringsjord and Schimanski (2003).

^38^In the case of the step presented in Figure 4, PERI.2 is able to utilize disjunctive syllogism to satisfy the probability query in schema [*I*_**K**_]. Essentially, because PERI.2 knows that there are already puzzle pieces in three of the four possible places it can put the blue piece, the piece must go in the only remaining place.

^39^This general premise is unfortunately far from implausible and is used as well in a simpler ARCADIA-less/perception-less adjudication scenario presented in Bringsjord et al. (2021), which is directly inspired by real events in the past.

^40^As the reader by now knows, DCEC has a perception operator (and a communication operator), but they are not in and of themselves connected to any genuine mechanization of attention and perception that is, in turn, based on the science of attention and perception in computational cognitive science. Connecting to ARCADIA changes this in one fell swoop.

^41^This agnosticism is, in part, based on the initial percepts of the people of interest in the garage.

^42^After all, program verification makes little sense if there cannot be programs that are invalid. For discussion of this, see Arkoudas and Bringsjord (2007). For a paradigm of program verification based directly on our brand of logicist AI, see Bringsjord (2015).

^43^That is to say, it determines and internally justifies whether ϕ follows from, or is contradicted by, first-order beliefs (i.e., its own beliefs about the world), as regulated by background inference schemata (which obviously include normatively invalid ones, e.g., affirming the consequent).

^44^Natural-language-generation aspects of TLM are left aside here since out of scope.

^45^Laid out e.g., in Bartha (2013); Bringsjord and Licato (2015).

^46^The three intensional prerequisites are: (i) Monty must *know* what's hidden by all doors; (ii) he must *perceive* and thereby come to *know* that initial choice; (iii) he must *intend* to open a losing door, and accordingly perform the associated action.

^47^We do not fully agree with Pollock's proposed solutions to this pair of paradoxes, but such matters are out of scope presently.

^48^Because (a) we momentarily provide information regarding how our approach will be extended into abductive reasoning, and (b) this information could not have been assimilated by the reader in advance of our laying out our approach, and instantiating it in the three case studies, we judged the present, concluding section to be the optimal location for our discussion of abduction. Notably, there are forms of abduction that in fact are not viewed as reasoning. This is nicely discussed in the study by Douven (2021), which begins with a key distinction: abduction viewed as the generation of hypotheses vs. abduction as the reasoning that justifies propositions, especially propositions that are hypotheses. Clearly, it is the latter form that is our concern.

^49^Because abductive reasoning is often described as “inference to the best explanation”, and such inferencing is (plausibly, in our opinion) taken by many to be a cornerstone of the empirical sciences (see Douven, 2021), more elaborate examples from science could be given instead of our simple parable, but doing so is beyond scope and available space here—but we provide a few leads: For the reader not all that familiar with abduction, but with logic and science, in general, our recommendation is to read a seminal abductive model from Hintikka (1998). For those with an interest like ours, i.e., in human-centric AI and cognition, the place to start is without question the recently released Magnani (2023), and for a somewhat older but still-relevant overview of AI and computational logic, see Paul (2000).
